# Parallel adaptation to geothermally-warmed habitats due to common structural variation and functional developmental pathways

**DOI:** 10.1038/s41467-026-75785-0

**Published:** 2026-07-22

**Authors:** Matthew K. Brachmann, Ana P. B. Costa, Shaun Robertson, Bethany Smith, Kate Donoghue, Natalie Pilakouta, Mark Whitehead, Xuan Liu, Bjarni Kristjánsson, Skúli Skúlason, Colin Selman, Kevin Parsons

**Affiliations:** 1 School of Biodiversity, One Health, and Veterinary Medicine, University of Glasgow, Glasgow, UK; 2 Marine Biology and Ecology, Rosenstiel School of Marine, Atmospheric and Earth Science, University of Miami, Miami, FL USA; 3 School of Biology, University of St. Andrews, Andrews, UK; 4 Liverpool Shared Research Facilities, University of Liverpool, Liverpool, UK; 5 Department of Aquaculture and Fish Biology, Hólar University, Saudárkrókur, Iceland; 6 School of Molecular Biosciences, University of Glasgow, Glasgow, UK

**Keywords:** Molecular evolution, Evolutionary developmental biology, Structural variation

## Abstract

Climate change is causing rapid temperature increases that drive adaptive changes. Predicting these outcomes remains challenging as the extremes of warming have not yet been experienced by natural populations. Contemporary populations inhabiting geothermally-warmed environments can provide valuable insights, including phenotypic and genomic responses involved in adaptation to warming. Here, we examine independent instances of divergence between geothermal and ambient ecotypes of threespine stickleback. While we identify similar patterns of phenotypic divergence in appetite, fat storage, and fat loss (during starvation), genomic divergence of loci is non-parallel. However, at a functional level, the *mapk* signalling pathway differs across ecotype pairs and expression of *mapk* genes differs between ecotypes, are thermally plastic, and influenced by hybridization. We also identify an inversion on chromosome XXI, previously attributed to freshwater colonization, diverging in parallel between ecotypes. Candidate genes within the inversion are associated with metabolic traits, including appetite regulation and fat storage in correspondence with our phenotypic findings. Overall, polygenic adaptation conferring functionally similar outcomes and structural variation are key genomic mechanisms facilitating adaptation to warmed environments. Functional molecular pathways should be prioritized in conservation, as precise allelic changes are difficult to predict and potentially less relevant to thermal adaptation than previously thought.

## Abstract (web summary)

Climate change is rapidly increasing temperatures, challenging species to adapt in place. Studying Icelandic stickleback, this study shows that adaptation to geothermally-warmed habitats involves both shared structural genomic variants and changes in key metabolic pathways.

## Introduction

Increasing global temperatures represent the most pervasive threat to biodiversity^1–3^. Ectotherms are predicted to be the most impacted by warming climatic conditions as the environment primarily regulates their body temperature^4–6^. This will be especially challenging for populations that are limited in their ability to disperse to new habitats, as they will either be forced to adapt in situ or go extinct^7^. Therefore, the capacity of organisms to adapt to increased temperatures, and the mechanisms behind this, will be crucial for predicting and mitigating biodiversity loss^8–10^. Thus, gaining insights into the genomic mechanisms underpinning thermal adaptation will play a critical role in mitigating climate change.

Rapid changes in climatic conditions are expected to drive adaptation through genomic mechanisms^9^. Many studies attempting to understand the genomic basis of climate change adaptation focus on changes in allele frequencies^8^. For example, Des Roches et al. (2020) showed that climate change over the last century is driving changes in allele frequencies of the *eda* gene in threespine stickleback (*Gasterosteus aculeatus*) across a latitudinal gradient. However, while specific genes are likely to be involved, temperature has broad effects on development, physiology, and in turn gene expression and function, making it likely that thermal adaptations will be polygenic and involve complex phenotypes^11,12^. Across multiple populations such polygenic traits will likely emerge though unique allelic variation and be impacted by genetic background (i.e. population specific epistasis), making generalizations about the genomic basis of climate change adaptation challenging. Indeed, allelic changes are often non-parallel across populations diverging along replicate habitat gradients^13^. Even systems with recently shared demographic histories across populations demonstrate a high degree of non-parallelism at the genomic level^14^. Importantly, natural selection will be blind to the specific loci involved with adaptation, with the ability to favour functional outcomes that improve fitness^15^.

Beyond allele frequency changes there is growing interest in other forms of genomic variation^9,10,12^. For example, the presence of structural variants, such as inversions, can allow organisms to adapt to changing environmental conditions^16^. Adaptation based on inversions is predicted to occur rapidly as they limit the impact of gene flow by restricting recombination thereby stabilizing links among combinations of adaptive alleles within a given haplotype block^17,18^. Indeed, large inversions can drive adaptation to contemporary environmental conditions and allow for the formation of different ecotypes^19–21^. Acting as a ‘supergene’ they can have a large effect on complex phenotypes^22^. Given the predicted speed of climate change, structural variants could promote rapid adaptation to changing thermal conditions although their role in thermal adaptation has rarely been identified^23–25^.

Predicting the evolutionary outcomes of climate change is an important challenge for genomics^8^. However, the extreme conditions predicted by climate change have not yet been experienced by wild populations. This has led researchers to compare populations from different latitudinal and altitudinal gradients^26,27^ and utilize genomic offset modeling to predict responses to climate change^28–31^. While valuable, comparing latitudinal gradients can impose confounding effects that influence evolution^32,33^ and interpretations of genomic offset models have critical limitations^34^. Conversely, lab experiments tend to involve responses that occur within a single generation rather than evolutionary change^35^. However, some extant populations can experience warming through geothermal activity and provide direct insights into responses to warming in natural systems. Geothermally warmed habitats should impose relatively strong selection on ectotherms as they are more metabolically demanding than ambient habitats, change developmental rates and processes, and offer novel ecological conditions^33,36^.

Here, we leverage Icelandic populations of threespine stickleback (*Gasterosteus aculeatus*) undergoing adaptation in response to thermal habitat variation imposed by geothermal warming^37^. The geothermal and ambient habitats primarily diverge in response to temperature as water chemistry and pH does not consistently differ between habitats^38^. However, geothermal and ambient habitats may vary in biotic factors such as resource availability and predation. These divergent populations exist in very close proximity, including cases of sympatry, and have repeatedly diverged between geothermal and ambient habitats throughout Iceland. This includes divergence in morphological^38^, metabolic^33^, and behavioural traits^39,40^. However, the genomic basis for such adaptive divergence across thermal habitats has not been investigated.

We assessed the genomic basis for thermal habitat divergence in these populations. First, we tested for the degree of genomic differentiation between geothermal and ambient ecotypes among populations across Iceland to gain a phylogeographic understanding of divergence. Second, using our understanding of wider geographic relationships, we selectively targeted populations to identify specific regions of the genome associated with parallel divergence between geothermal and ambient habitats. Third, we aimed to assess the functional and adaptive significance of genes differentiating geothermal and ambient ecotypes including phenotypic and genomic approaches. We identified a wide range of genetic variation involved with thermal habitat divergence, which included developmental and metabolically relevant genes. A particularly striking finding was a structural variant (ancestral inversion) differentiating geothermal and ambient ecotypes in parallel across all populations. This putative inversion contains multiple metabolically important genes and appears to regulate appetite, fat stores, and starvation responses in geothermal environments.

## Results and Discussion

### Phylogeographic and population genetic structure

**Fig. 1:**
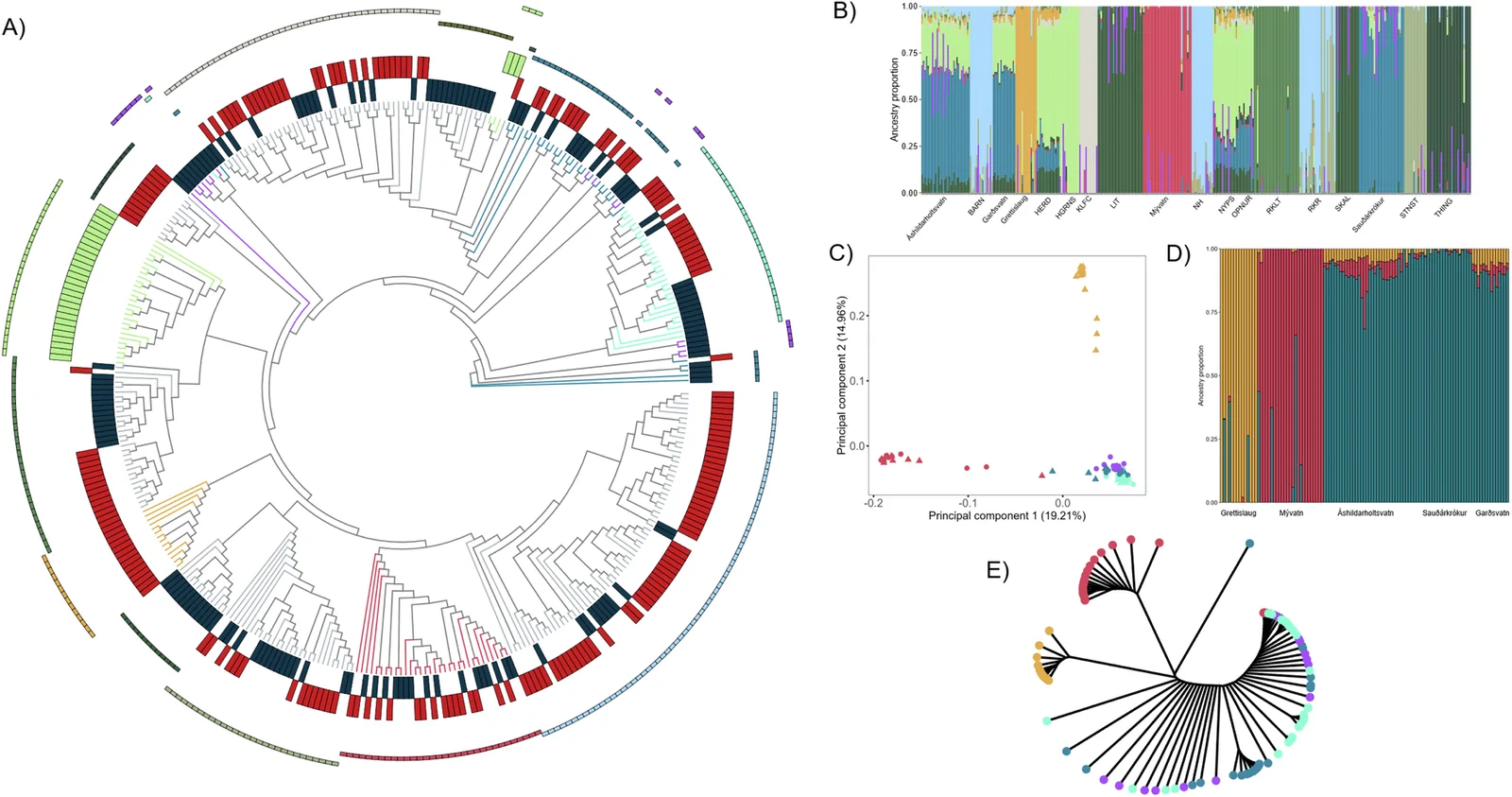
Population genetic relationships between geothermal and ambient ecotype pairs of Icelandic threespine stickleback. **A** Phylogenetic relationships between 25 stickleback populations based on genotype-by-sequencing (GBS). The unrooted neighbor joining tree used a Balanced Minimum Evolution (BME) iterative addition method with Subtree Pruning and refinement (SPR) tree refinement. The coloured branches represent the populations chosen for whole genome sequencing. The red, blue, and green middle ring respectively shows the distribution of geothermal, ambient, and marine ecotypes. The outer ring shows the grouping by geographical region based on the respective latitudes and longitudes of each lake (Table S1). **B** Admixture between the 25 stickleback populations estimated using ngsADMIX where the optimal k-value was 11, based on highest likelihood. Each color relates to an admixture group. **C** Principal component analysis (PCA) showing the relationships between GTS (orange), GAR (purple), ASHN (dark blue), SKR (light blue), and MYV (red) and the geothermal/ambient ecotypes within them. Geothermal and ambient ecotypes are shaped as triangles and dots, respectively. **D** Admixture analysis showing genetic admixture across the five stickleback populations (*K* = 3). **E** Phylogenetic relationships among GTS (orange), GAR (purple), ASHN (dark blue), SKR (light blue), and MYV (red) populations estimated using UPGMA on a whole genome distance matrix.

Our results highlighted that stickleback populations were clearly differentiated by geography, in line with previous studies across environmental gradients^41^. Generally, each freshwater body formed its own branch on the distance based phylogenetic tree (Fig. 1A). However, five sites (Sauðárkrókur (allopatric geothermal and ambient ponds), Garðsvatn (ambient lake), and Áshildarholtsvatn (sympatric geothermal and ambient sites) within the Sauðárkrókur region of northern Iceland) clustered together, despite having varied sympatric and allopatric demographic histories, likely because they are in close physical proximity to one another making shared ancestry likely (Fig. 1A–E; Suppl. Fig S1). Despite being geographically separated, the two marine populations were genetically similar to each other, indicating a panmictic marine population acting as a reservoir of standing genetic variation^42,43^. Many of the freshwater populations were likely colonized directly from the marine environment ~10,000 years ago during the deglaciation of Iceland^44–46^ and many populations show ancestry with marine stickleback (Fig. 1B). However, we also included water bodies formed within the last 50–70 years that would have likely been colonized from other freshwater populations of stickleback^45^. The oldest location (Grettislaug) was the most differentiated from all other freshwater populations and may have been one of the earliest sites colonized by marine stickleback among our populations. Common patterns of genomic divergence despite large differences in population age, relationships, and demographic history would indicate robust divergent selection between geothermal and ambient habitats. Therefore, we selected sites for further investigation that represented old (> 2000 ypb, Mývatn (MYV), Grettislaug (GTS)/Garðsvatn (GAR)) and young populations (~70 ybp, Áshildarholtsvatn (ASHN), Sauðárkrókur (SKR)) as well as allopatric (GTS/ GAR, SKR and sympatric (MYV), (ASHN)) conditions.

For these populations, whole-genome resequencing revealed a high degree of variation in admixture among sites. We identified three distinct clusters (*K* = 3 corresponding to GTS, MYV, and sites in the Sauðárkrókur region) across Iceland encompassing geothermal and ambient ecotypes diverging in both sympatric and allopatric conditions (Fig. 1C–E). Among these clusters there was little admixture; however, the populations from the Sauðárkrókur region show a low degree of population structure (Fig. 1C–E). The pattern of population structure is more than likely due to the colonization/isolation age of the freshwater lakes, where GTS and MYV were more than likely colonized earlier than the populations in the Sauðárkrókur region. The lack of population structure may promote shared genomic variation (alleles or structural variants) across populations and parallel genomic adaptation to contemporary environments^47,48^.

### Local adaptation to geothermal habitats is polygenic

Genome wide genomic differentiation (Fst) between geothermal and ambient ecotypes was low, but varied, across all populations (MYV = −0.0022; ASHN = 0.0027; SKR = 0.012; GTS-GAR = 0.050). Allopatric sites had greater genomic differentiation between ecotypes than sympatric sites, likely due to a lack of gene flow and promotion of genetic drift^49–52^. While reduced gene flow does aid in promoting increased genomic differentiation, differences in thermal habitat appeared to be the driver of genomic differentiation between ecotypes across all population pairs.

**Fig. 2:**
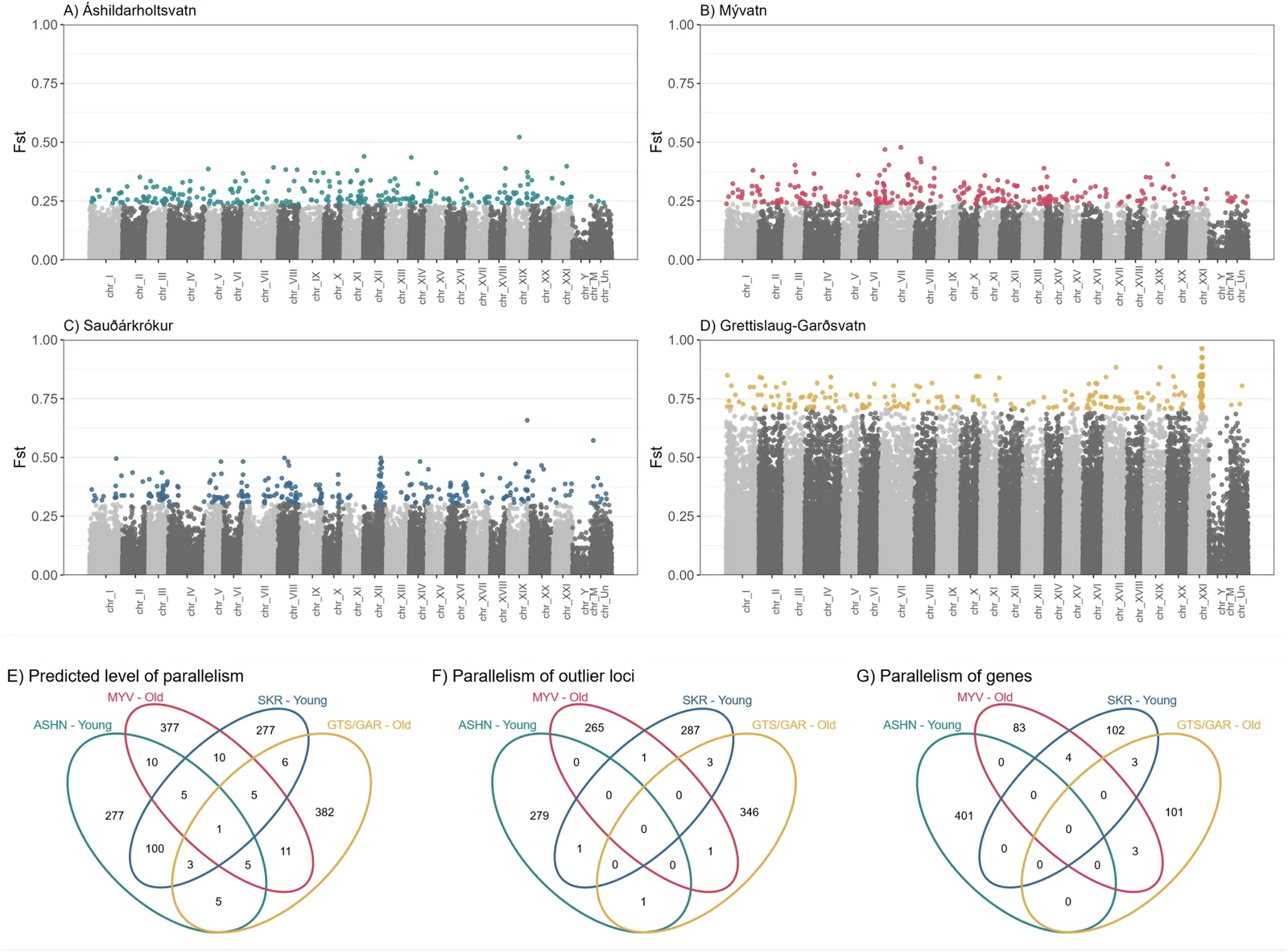
Population genomic differentiation between geothermal and ambient ecotypes. **A**–**D** Per locus population differentiation (Fst) across the stickleback genome for four independent geothermal/ambient ecotype pairs. Neutral loci are coloured as shades of grey while Fst outlier loci are coloured according to the ecotype pair. Fst outlier loci were in the top 0.5% of the Fst distribution. **E** Predicted degree of genomic parallelism across ecotype pairs based on geography and time since divergence. We provide a predicted level of genomic parallelism if population age and time since divergence had an effect on the number of shared outlier loci and genes (based off of^173^). If time since divergence plays a role in structuring the number of outliers across populations, younger ecotype pairs would share more outliers with each other than older ecotype pairs. The older ecotype pairs would share some outliers with each other but have less overlap in general due to adaptation, drift, and the erosion of genomic variation due to a longer time since divergence. **F** Parallelism of outlier loci differentiating geothermal and ambient ecotype pairs. **G** Parallelism of divergent genes between geothermal and ambient ecotype pairs. All colours in (**E**–**G**) correspond to the Manhattan plots in (**A**–**D**).

Indeed, despite low overall genomic differentiation between ecotypes across population pairs, we detected extensive per-locus genomic differentiation between thermal ecotypes regardless of population age and demographic history. Genomic differentiation between thermal ecotypes is highly polygenic involving hundreds of outlier loci (ASHN = 281; MYV = 267; SKR = 292; GTS-GAR = 351) across multiple chromosomes (Fig. 2A–D; Supp. Figs. S5, 6). This supports the idea that polygenic adaptation for complex adaptive phenotypes is more common than previously thought^53^. We also detected regional Fst peaks differentiating geothermal and ambient ecotypes across the genome (Fig. 2A–D). However, there was a low degree of parallelism in Fst outlier loci, compared to predicted levels of parallelism (Fig. 2E), across geothermal and ambient ecotype pairs (Fig. 2F; 95% confidence interval: 0.00–0.00024, *p*-values < 0.001; Table S2). We found no relationship between neutral divergence and parallelism in outlier loci (F-value: 0.54, R² = 0.11, *p* = 0.87), indicating that differences in divergence time do not explain the limited overlap in outlier loci. Notably, the youngest (SKR, ~70 generations) and the oldest population pairs (GTS-GAR, ~4000 generations) shared the greatest overlap in Fst outliers (3 shared Fst outliers), indicating that variation in age did not provide a strong influence on thermal habitat divergence.

Consistent with low parallelism in outlier loci, we also detected low parallelism across ecotype pairs at the gene level (Fig. 2G; 95% CI: 0.00–0.00012, *p*-values < 0.001; Table S2). We found no relationship between neutral divergence and parallelism at the gene level (F-value: 0.57, R² = 0.12, *p* = 0.58), indicating that divergence time does not explain the limited overlap at this level. Genomic parallelism in cases of divergent parallel phenotypes across populations has been shown to increase at functional levels of the genome^14,54^. This contrasts with previous findings that stickleback ecotypes diverging across other environmental gradients have a high degree of parallelism at a single locus^55,56^ as well as multiple loci^57,58^. The genomic architecture of polygenic adaptation to thermal habitats is largely non-parallel across independent ecotype pairs. However, low genomic parallelism does not preclude the possibility that selection favours similar biological function. Indeed, signalling pathways provide opportunities for genetic redundancy and can lead to highly parallel molecular pathway function and ultimately parallel phenotypes^59–62^.

The low degree of genomic parallelism indicates a complex and polygenic genomic basis for thermal phenotypes. This is supported by our findings of highly polygenic genome-wide differentiation as the average number of genes differentiating geothermal and ambient ecotypes was 99 (ASHN = 93; MYV = 90; SKR = 109; GTS-GAR = 107). Between 34.3 and 45.5% of outlier loci were located within non-coding regions (ASHN = 38.4%; MYV = 37.3%; SKR = 42.5%; GTS-GAR = 34.2%) while 17.6% to 24.2% were within protein coding regions of exons (ASHN = 17.6%; MYV = 16.5%; SKR = 19.3%; GTS-GAR = 24.2%). A low proportion of outlier loci were located within promoter regions (5̕ untranslated region) (ASHN = 0.93%; MYV = 0.97%; SKR = 0.81%; GTS-GAR = 1.7%) and 3̕ untranslated regions (ASHN = 0.93%; MYV = 1.94%; SKR = 0.81%; GTS-GAR = 0.83%). Much of the genomic differentiation between ecotypes appears to be driven by non-coding and potentially regulatory regions of the genome, a finding in line with previous comparisons between marine and freshwater stickleback where only 17% of divergent SNPs were within coding regions of genes (Jones et al., 2012). Changes in regulatory regions in genes associated with adaptive divergence may be more prevalent than previously thought and may allow for the divergence of complex genomic networks and cellular pathways^63,64^. However, the rates of evolutionary change are typically increased in either regulatory or coding regions but rarely both simultaneously^65^. Given the large-scale of polygenic changes to regulatory functions of genes here, as well as the non-parallel nature of adaptive divergence to similar thermal habitats, we suggest that predicting allelic responses to increased warming will not be tractable^66^. Thus, gene-targeted conservation strategies will likely fall short of conserving species under changing climatic conditions^67^. Accordingly, conservation genetic/genomic strategies should consider polygenic models of adaptation and target functional molecular pathways capable of producing adaptive phenotypes.

Our gene network analyses for genes containing outlier loci for each geothermal and ambient ecotype pair identified a putative candidate pathway (Suppl. Table S3; Suppl. Figs. S2–4). Of particular interest were genes associated with the *mapk* signalling pathway which appeared prominently across ecotype pairs and included specific *mapk* genes (*map2k5*in ASHN; *mapk1* in MYV and SKR; *mapk13* in SKR; *myb* and *ppp3cb*in GTS-GAR). The *mapk* signalling pathway has been implicated in cell proliferation and differentiation as well as neural crest cell development^68^ and in turn craniofacial development^69,70^. This matches with our previous findings of parallel morphological divergence in the craniofacial apparatus across ecotype pairs^38^. Such morphological divergence is both heritable and adaptive across thermal habitats^37,38^. While neural crest cells have been widely reported as a mechanism underlying adaptive variation^71^, it is notable that they are also sensitive to temperature variation with activated ion channels conferring maternal fever-associated birth defects^72^. Environmental variation in temperature can facilitate differences in the timing of neural crest cell differentiation and proliferation as well as differences in cell type differentiation^72^. Thus, while more investigation is required, neural crest cells may play a major  role in thermal adaptation.

### The functional role of *mapk* gene expression in geothermal and ambient ecotypes

**Fig. 3:**
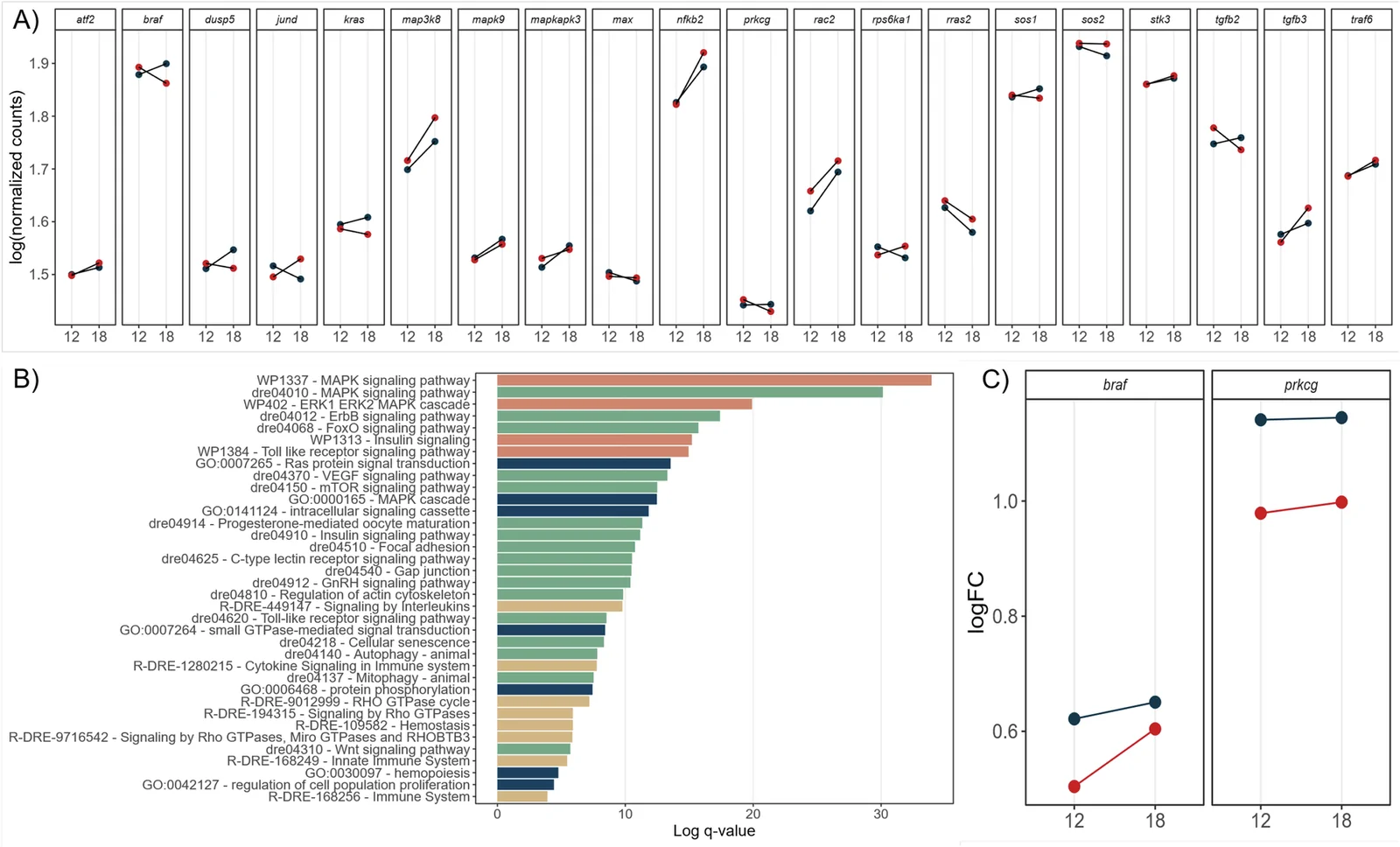
Expression of genes in the mapk pathway. **A** Mean gene expression profiles from a common garden experiment where each gene had a significant ecotype-temperature interaction. Red and blue colours represent geothermal and ambient ecotypes, respectively. Gene expression was assessed using a GLM with population pair as a fixed effect. **B** Gene ontology (GO) results from all *mapk* genes that had a significant ecotype-temperature interaction. The different colours correspond to the different databased used for the GO analysis. **C** Mean gene expression profiles from a common garden experiment from geothermal, ambient, and hybrid ecotypes from the SKR population. Red and blue colours represent geothermal-hybrid and ambient-hybrid divergence at 12 and 18 degrees, respectively. The two *mapk* genes depicted overlapped as outliers between the previous common garden experiment and show transgressive expression patterns relating to hybridization.

Our common garden experiments suggested that the *mapk* signalling and developmental pathways are under divergent selection between geothermal and ambient ecotypes. We identified 20 genes within the mapk signalling pathway with significant differences in gene expression (*p*-value < 0.05) related to an ecotype by temperature interaction (Fig. 3A). Gene expression of *mapk* genes was divergent between geothermal and ambient ecotypes at each rearing temperature, and this pattern of divergence was plastic in response to temperature, indicating heritable and divergent plastic responses between ecotypes^73^. These genes were primarily associated with the *erk 1/2 mapk* pathway based on a gene ontology (GO) analysis (Fig. 3B). Divergence in thermal plasticity between geothermal and ambient ecotypes suggests that the *erk 1/2* signalling pathway, and the *mapk* pathway more broadly, plays a role in adaptation to geothermal and ambient environments^74,75^. Indeed, gene regulatory changes in the *mapk* pathway have previously been shown to facilitate metabolic adaptation to warming environments^76–79^.

Hybridization may disrupt adaptive gene regulatory architectures and produce less fit hybrid offspring which both facilitates and reinforces reproductive isolation between ecotypes^80^. In a separate common garden experiment, we identified two *mapk* genes (*B-Rapidly Accelerated Fibrosarcoma (braf)* and *Protein Kinase C gamma* (*prkcg*)) that showed transgressive expression in hybrids from the SKR population (Fig. 3C). Transgressive patterns of gene expression occur when hybrids have greater or lower gene expression relative to the non-hybrid ecotypes^75,81^. In this case, there was higher expression of *braf* and *prkcg* in hybrids relative to geothermal ecotypes in 18˚C rearing environments, but there were no changes in hybrids relative to ambient ecotypes across rearing environments. *Braf* regulates cell growth, division, and differentiation and is activated through the insulin signalling pathway^82–84^, while *prkcg*, part of a class of protein kinases with a diverse set of functions, appears to be at least partially regulated by hyperglycemia^85^. The *braf* gene showed elevated Fst between ASHN and MYV wild geothermal and ambient ecotypes (ASHN = 0.03, MYV = 0.005) but not in SKR and GTS-GAR (SKR = 0.08, GTS-GAR = 0.06), suggesting that the *braf* gene might be under selection in wild populations^50,86–88^. Geothermal and ambient ecotypes have likely evolved different gene regulatory architectures in the *mapk* pathway, whereby recombination of these regulatory architectures during hybridization produces novel phenotypic expression^75,81^. Transgressive expression of key *mapk* genes, such as *braf*, may lead to reduced hybrid fitness^80,89^ as decreased expression, as seen in geothermal ecotypes under 18˚C, can decrease glycolysis in the cell^82,83,90^ and indicates a switch to an alternative metabolic pathway such as lipid metabolism^91,92^. Hybrids may be unable to switch to alternative metabolic pathways when raised in warm water conditions which is likely to be maladaptive in geothermal environments. While the *mapk* signalling pathway appears to facilitate adaptation to geothermal habitats, further functional validation of the effects of the pathway on phenotypic variation are required.

### Parallel divergence between geothermal and ambient habitats relies on structural variation

**Fig. 4:**
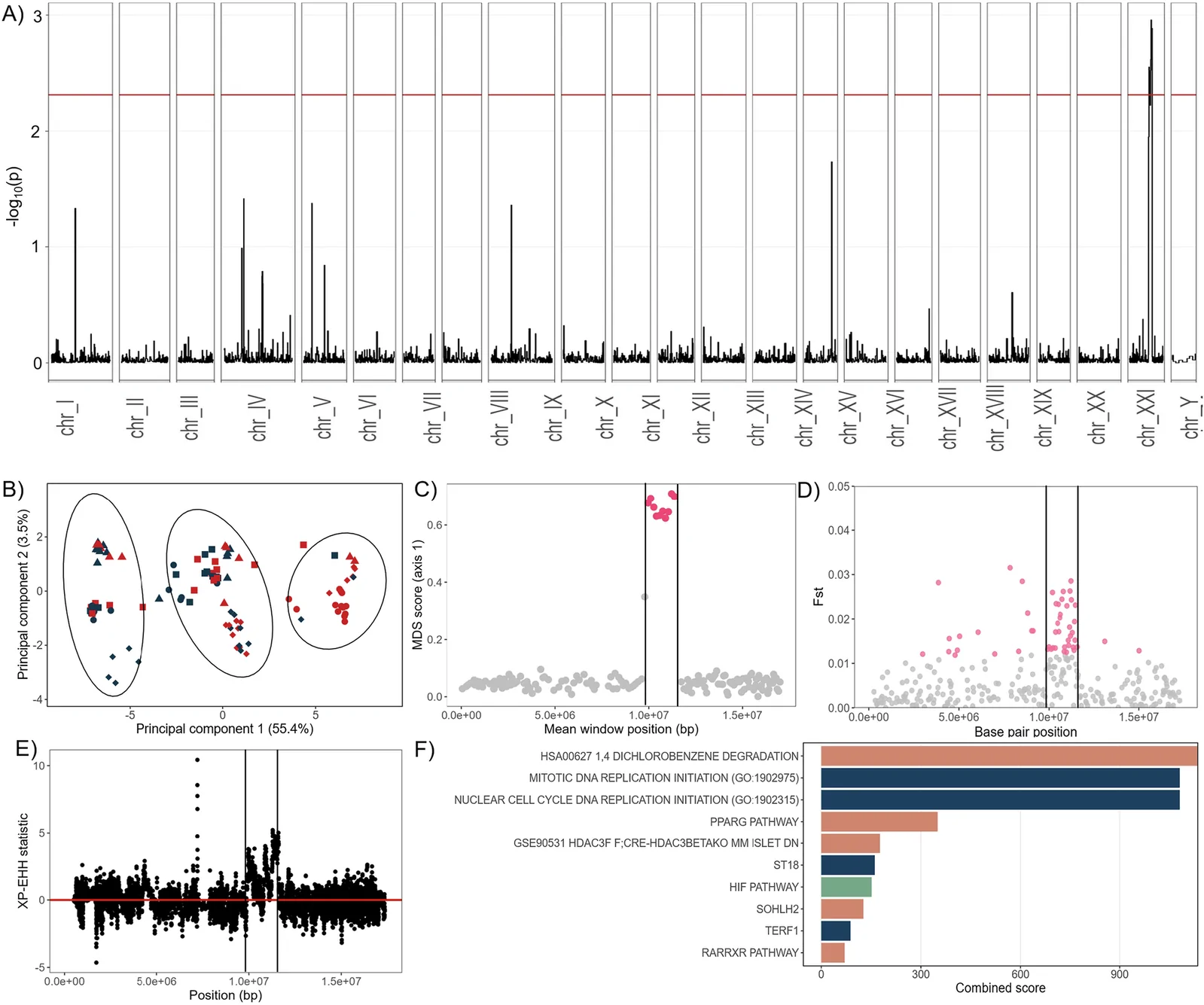
Assessment of genomic variation differentiating geothermal and ambient ecotype pairs in parallel across replicate populations. **A** Parallel differentiation of allele frequencies, in 50 SNP windows, between geothermal and ambient ecotype pairs (AF-vapeR). The red line indicates significance based on a null distribution of allele frequencies. Significant peaks of parallel differentiation in allele frequencies are located on chromosome XXI. **B** Principal component analysis (PCA) of the region differentiating geothermal and ambient ecotypes in parallel. Red and blue colours represent geothermal and ambient ecotypes. Ellipses highlight groups based on k-means clustering. Shapes represent the different geothermal-ambient ecotype pairs. **C** Differentiation of population structure between geothermal and ambient ecotype pairs along chromosome XXI in 50 SNP windows (lostruct). Significant deviations in population structure are coloured while neutral windows are represented in grey. Deviations in population structure using local PCA have been previously indicative of structural variants. **D** Per locus differentiation between all geothermal and ambient ecotype pairs. Outlier loci are coloured while neutral loci are represented in grey. **E** Putative selective sweeps between geothermal and ambient ecotype pairs along chromosome XXI. The red line indicates neutrality, positive deviations indicate selective sweeps in geothermal ecotypes, negative deviations indicate selective sweeps in ambient ecotypes. **F** Identification of biological functions for the genes within the region of interest on chromosome XXI using gene ontology (GO). The colours correspond to different biological functions (blue = DNA and cellular repair, green = hypoxia, and orange = metabolism).

Despite low levels of genomic differentiation (interspersed with outliers) we found strong evidence of parallel differentiation in a localized but multigene region. Specifically, a 1.6 megabase (Mb) region on chromosome XXI (9963830-11574024 base pair position) showed both elevated and common allele frequency divergence between thermal ecotypes (Fig. 4A; Suppl. Fig. S5–8). A principal component analysis (PCA) on the entire 1.6 Mb region (Fig. 4B; Suppl. Fig. S9), as well as a windowed local PCA (Fig. 4C), show the characteristic population genomic structure of an inversion^20,93^. In the PCA of the entire region, the majority of geothermal individuals were in the inverted homokaryotype cluster suggesting that this inversion is associated with temperature adaptation. Within the 1.6 Mb inversion region there was a distinct Fst peak differentiating thermal ecotypes (Fig. 4D). Overall, divergence was relatively high with 46.2% of all loci on chromosome XXI being Fst outliers, and with 64% of these loci being located within the 1.6 Mb region. This divergent region of chromosome XXI showed elevated levels of the XP-EHH statistic (Fig. 4E) suggesting strong recent selection associated with the geothermal environment. The combination of these results indicated that the geothermal karyotype is more than likely the derived state and inverted karyotype as it is under selection.

Structural variants have been implicated in promoting adaptive divergence among ecotypes as large regions of co-adapted genes can be inherited as a single unit^12,94^ and thus effectively act as a single locus that is resistant to the effects of recombination^17,19,95^. The structural variant we have identified on chromosome XXI has been previously identified as an inversion associated with the transition to freshwater environments from marine environments in threespine stickleback^62^. However, the functional impacts of the inversion on chromosome XXI are unknown as it does not appear to be related to salinity as suspected^96,97^. As our stickleback all originated from freshwater environments, we instead suggest this inversion is related to transitions from cold to warm thermal habitats across our populations and for the species as a whole. Marine and freshwater environments differ not only in salinity but also temperature profiles as freshwater habitats are generally more variable and achieve warmer temperatures than marine habitats^98,99^. Wild marine and freshwater stickleback differ in their ability to handle extreme temperatures as marine individuals have impaired cold tolerance relative to freshwater individuals^100^. However, while cold tolerance can evolve rapidly (within three generations) heat tolerance does not, suggesting a more complex genetic basis for adaptation to warmer temperatures. Therefore, understanding the effects of this inversion on thermal habitat divergence could provide more insight into the function of inversions, including the speed at which thermal adaptation can occur, as it is implicated in both young and old population pairs.

The putative inversion contained genes relevant to thermal adaptation, including metabolism, hypoxia, and cellular repair (Fig. 4F). Gene ontology analysis on the 55 genes in this region matches with our previous findings implicating morphology and metabolism as divergent traits in these systems^33,38^. More broadly for threespine stickleback, previous independent studies have identified 23 morphological QTL within this region that are associated with feeding, body shape, defence, and sensory systems^101–104^. Geothermal environments are stressful and promote strong selection for adaptations which increase performance under stress. Thermal stress can induce DNA damage and cellular apoptosis^105,106^. DNA and cellular repair mechanisms may be needed to repair deleterious genomic mutations induced by living in geothermal environments. Geothermal environments have also been shown to induce elevated metabolic rates in ectothermic organisms^5^. For metabolism in particular, the geothermal habitats should cause long periods of low resource availability during winter^45,107^, but alongside elevated metabolic demands due to higher temperatures. The seasonal metabolic demands faced within geothermal habitats are similar to the seasonal metabolic demands shown to influence divergence in Mexican cave fish (*Astyanax mexicanus*)^108^. Mexican cave fish have shown remarkable adaptation to seasonal resource limitation through changes in a suite of metabolic processes^109–112^. While speculative, the convergent metabolic signatures across diverse systems may involve similar physiological mechanisms. The 1.6 Mb inversion located on chromosome XXI potentially enables enhanced DNA repair and metabolic adaptation to thermal stress under geothermal conditions.

### Connecting adaptive phenotypes to candidate genes

**Fig. 5:**
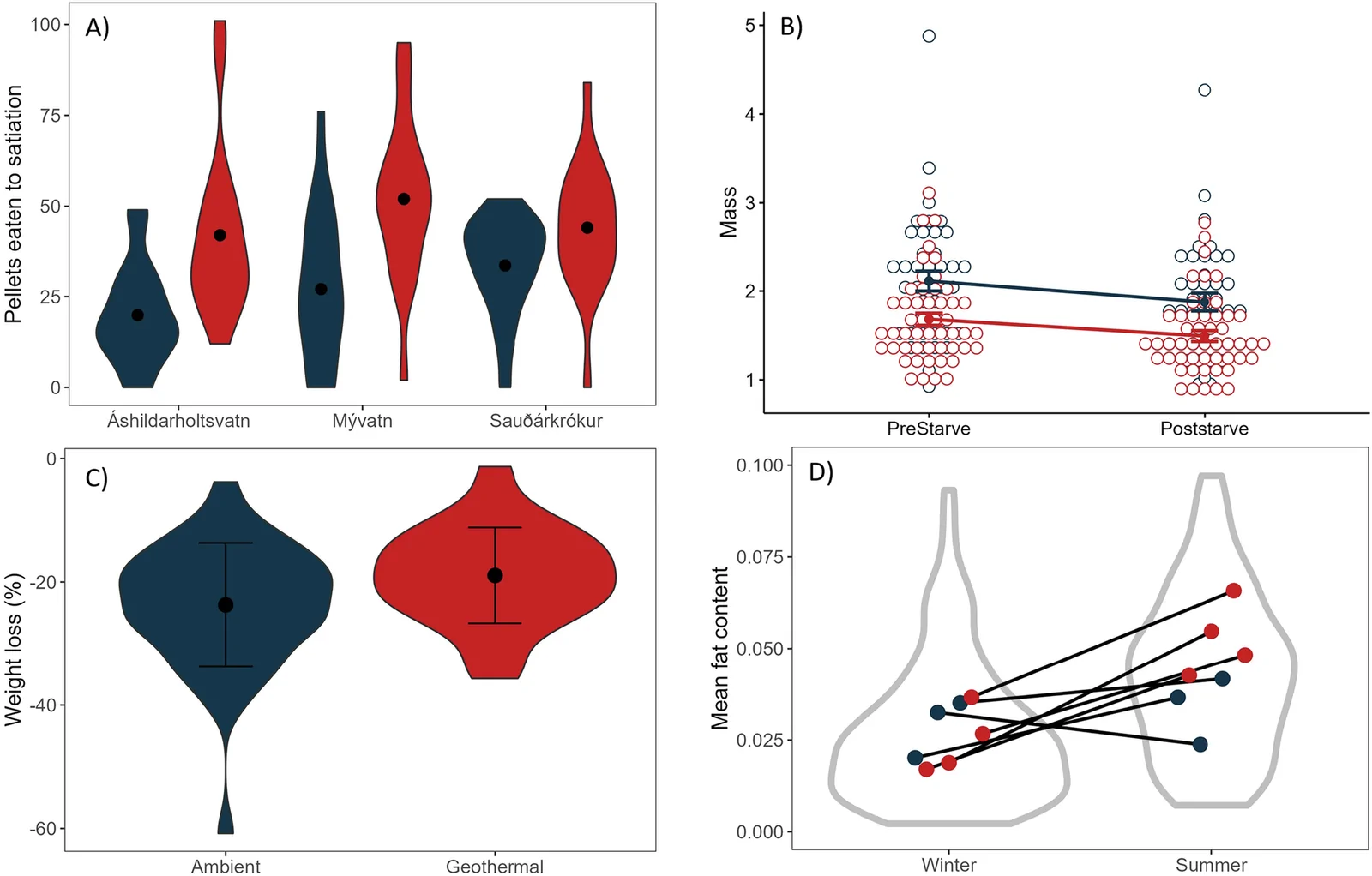
Phenotypic differences between geothermal and ambient Icelandic stickleback ecotypes. Geothermal and ambient ecotypes are shown in red and blue, respectively. **A** Differences in appetite phenotypes in F2 progeny shown by the number of pellet food eaten from ASHN, MYV, and SKR. The mean number of pellets eaten is indicated by the black dot. **B** Mass differences between geothermal and ambient ecotypes from ASHN and MYV pre- and post-starvation for 30 days. Error bars indicate standard error surrounding the mean estimates. **C** Percent weight loss post-starvation for geothermal (red) and ambient (blue) ecotypes from ASHN and MYV. Points represent the mean and the error bars represent standard deviation. **D** Fat content of geothermal and ambient ecotypes from four Icelandic ecotype pairs between summer and winter seasons. Points are represent the mean fat content for each ecotype across the two season and the violin plots show the distribution of fat content between the seasons across all ecotypes.

While several candidate genes for geothermal/ambient divergence exist within the putative inversion region, some were of particular interest. This included the presence of *Nuclear Receptor Coactivator 2* (*ncoa2*), which has been linked to appetite regulation^113–116^ and lipogenesis^114^. In our GO analysis on the genes in the inversion, *ncoa2* was related to the *Peroxisome Proliferator Activated Receptor* pathway (*ppar*) and occurs through a regulatory process where *ncoa2* binds to *ppara* as constituent parts of the pathway associated with appetite and lipogenesis^115^. *Ppara* has previously been shown to regulate lipogenesis in Mexican Cavefish^110^ as part of an adaptive strategy to alleviate long periods of low food availability. Thus, an *ncoa2/ppara* interaction could be regulating appetite levels and lipogenesis/obesity in geothermal ecotypes. The *ppar* pathway is well known to interact with the *mapk* pathway^117–120^ and work together to promote metabolic adaptation through lipogenesis^121^. While we have not directly tested this potential link, we have found strong heritable differences in appetite between ecotypes where geothermal fish exhibit hyperphagia with much higher appetite levels than ambient ecotypes (ASHN = 53.9% greater; MYV = 63% greater; SKR = 35.8% greater appetite) (DF = 1, F-value: 27.40, *p*-value = < 0.001, Fig. 5A; Suppl. Fig S10). Lipogenesis also seems to differ between ecotypes as geothermal fish lose mass at a slower rate than ambient ecotypes when food intake is reduced (DF = 1, F-value = 6.47, *p*-value = 0.013) (Fig. 5B, C). Geothermal ecotypes also increase fat content more rapidly during the summer season relative to ambient fish (Fig. 5D). Due to logistical constraints different population combinations were used across assessments of appetite and body fat (between seasons and under dietary restriction) but, three divergent populations (MYV, ASHN, and SKR) appeared in all experiments providing replication. Despite this limitation, we found consistent differences in appetite and amount of body fat (between seasons and under dietary restrictions) between geothermal and ambient ecotypes regardless of their population of origin. *Ncoa2* variants and their interaction with *ppara*, mediated by the *mapk* pathway, are potential candidates regulating increased appetite (hyperphagia) and lipogenesis in the geothermal ecotypes. Hyperphagia could be adaptive in geothermal fish leading to increased lipogenesis and energy reserves for winter periods of low food availability while under high metabolic demands due to warm temperature. Hyperphagia has previously shown to be adaptive in Mexican cavefish as a means to bridge periods of resource limitation^122^ while increased appetite has been linked to obesity in most vertebrates^123^.

### A way forward for evolution: the interaction of polygenic adaptation and structural variation

Over the last two decades, researchers have searched for the parallel genomic basis of adaptation to novel or changing environments with limited success^13,124–127^. This likely speaks to the complex polygenic nature of adaptation, with the involvement of many genes of small to moderate effects. However, large structural variants are likely to be different as they can be comprised of several genes with varying phenotypic effect sizes, while the lack of recombination they impose essentially causes them to be inherited as a single entity^128^. Thus, such structural variants, when present in a common ancestor, are likely to promote parallel genomic adaptation from standing genomic variation^18^. This is because structural variants, such as inversions, may be at low frequency in ancestral populations but, because of their large phenotypic effects can be easily ‘seen’ by natural selection to rapidly rise to high frequency^129^. This is similar to observations from genes of large phenotypic effects where major loss of function mutations lead to rapid evolutionary change, and seem to be more frequently exhibit genetic parallelism^130–132^. In this scenario, initial colonization and subsequent strong selection can provide rapid change on the basis of structural variants, following this lead and coarse-grained adaptation further refinement occur with selection modifying developmental pathways that interact with those disrupted by the major effect structural variant. Importantly, this refinement involves genome-wide polygenic adaptation that arises from standing genomic variation after the initial colonization event and becomes phenotypically expressed in the new environment in response to the structural variant. This secondary process is likely to increase the degree of non-parallel genomic differentiation, but with functional parallelism, across replicate populations as each population has undergone independent demographic processes^51^. Here, we identified a large structural variant on chromosome XXI that promotes parallel adaptation to geothermal habitats across replicate populations spanning different demographic histories and ages. Genes within this structural variant have been shown to influence the function of the *mapk* pathway^117,121,133^, that we found differentiates geothermal-ambient ecotype pairs through a non-parallel genomic basis. In short, structural variants, like genes of major phenotypic effect, could promote rapid adaptation to changing or novel environments enabling persistence and refinement through selection resulting in functional equivalency through a non-parallel genomic basis. To our knowledge, interactions between structural variation and polygenic adaptation have not been demonstrated but should be an area of future research.

Warming of natural habitats will be of increasing concern over the next decade. Here, we have provided direct evidence for a range of genomic responses to changes in thermal habitat variation in a series of natural populations of threespine stickleback. Overall, genomic differentiation between geothermal and ambient ecotypes is generally non-parallel at specific loci, but specific gene families (in this case *mapk* genes) provide functional similarity in molecular pathways across independent populations. However, a large putative inversion shows strong evidence of parallel divergence and involves genes relevant to a range of adaptive phenotypes. Particularly, within this region *ncoa2* provides a logical candidate for divergent appetite levels and lipogenesis between geothermal and ambient ecotypes. Together, these findings demonstrate that climate change adaptation is likely to involve complex genomic changes that are largely population-specific. While geothermal warming currently exceeds near-term climate change projections, developmental pathways like the *mapk* pathway may represent conserved targets of thermal adaptation. Thus, targeting certain genomic changes may fail to conserve species in warming environmental conditions, but an approach focused on conserving particular molecular pathways may be more efficient^60^. Structural variants, like inversions, may be more prevalent than previously thought in promoting adaptive divergence in this context and may help to mitigate biodiversity loss due to climate change. The complexity of such genomic changes may pose issues of tractability for conservation genomic management. Investigating the relative influence, and interactions, between structural variants and allelic variation on phenotypic adaptation may provide some resolution.

## Methods

All animals were housed and utilized in accordance to the Home Office regulations of animal welfare in this research conducted at the University of Glasgow (PPL3299715). Animal welfare oversight was provided by the Named Animal Care and Welfare Officer (NACWO) and Named Veterinary Surgeon (NVS) at the University of Glasgow Zebrafish Research Facility (ZRF). Import of stickleback from Iceland to the University of Glasgow was approved by Marine Scotland.

### Study sites and sampling

To gain a phylogeographic understanding of the genetic variation occurring across populations, threespine Stickleback were collected from twenty-three freshwater populations and two marine populations across Iceland (*n* = 365) (populations were defined based on geographic location of sites). Geothermal habitats generally had temperatures greater than 20°C, while ambient habitats were roughly 10°C colder within the summer months^38^. Freshwater populations were also classified as sympatric or allopatric populations depending on the degree of connectivity between geothermal and ambient locations. Sympatric populations allowed for continuous migration or gene flow while allopatric populations had a physical barrier separating geothermal and ambient habitats. Sticklebacks were sampled between May and June in 2016 and 2017 using minnow traps. Fish were humanely euthanized, and fin clips taken were placed in 100% ethanol. All fish were handled following UK Home Office guidelines for animal use.

### Genotyping-by-sequencing to determine phylogenetic relationships

The genomic DNA was extracted from fin clips taken from all individuals sampled using either the Qiagen DNeasy blood and tissue kit or the Applied Biosystems MagMAX DNA Multi-sample kit used on the Kingfisher Flew automated DNA extraction machine following the manufactures protocols. Quality of the extracted DNA was assessed using a Nanodrop 8000 spectrophotometer and a 1% agarose gel while DNA concentration was measured using a Qubit BR assay in a Qubit 2.0 Fluorometer.

Genotype-by-sequencing (GBS) library preparation was conducted using the genomic DNA of all stickleback samples. GBS sequencing libraries were constructed following the protocols outlined in ref. ^134^ with some modifications. A total of 100 ng of genomic DNA was digested with ApeKI and custom adapters ligated to the resulting sequence overhang. The custom adapters were designed using a dual indexing approach to control for index hopping. Samples were pooled and a double-sided size selection step was performed with AMPure AP beads using a bead:sample ration of 0.7:1 followed by a 1:1 ratio for an insert size range of 100–400 basepairs (bp). Polymerase chain reaction (PCR) was performed in a 50 μl reaction with 25 μl NEBNNext Ultra II mastermix, 25 μM of each primer, and 20 μl of pooled library. The PCR profile was 30 s at 98 degrees Celsius followed by 5 cycles of 10 s at 98 degrees Celsius, 30 s at 70 degrees Celsius, 45 s at 65 degrees Celsius, with a final extension of 5 min at 65 degrees Celsius. Libraries were analysed on an Agilent Bioanalyzer High-Sensitivity Chip for quality control. Paired-end sequencing with 150 bp read length was conducted on an Illumina HiSeq 4000 sequencer at the Center for Genomic Research, University of Liverpool.

Raw reads were de-multiplexed with GBSX v1.3^135^ using the unique dual indices per sample. Any reads not containing the possible barcode combinations were removed. FastQC v0.11.7^136^ was used to determine the quality of the sequence data. The reads were filtered and trimmed using Trimmomatic v0.36^137^. Leading and trailing bases were set to 3, the minimum Phred score was set to 20, the sliding window to 4 bp, and reads less than 30 bp were removed. Reads were aligned to the Stickleback reference genome^138^ using BWA-MEM v0.7.17^139^. The alignment bam files were sorted and indexed with Samtools v1.6^140^. Samples were re-aligned around indels using GATK v4.1.3.0^141^ with the functions RealignerTargetCreator and Indelrealigner using their default parameters. Quality of the mapped reads were checked with the functions flagstat and depth in Samtools, and samples that did not pass the quality-check were removed from downstream analyses.

Biallelic single nucleotide polymorphisms (SNPs) were called using ANGSD v0.929^142^. ANGSD allows for calculating genotype likelihoods for each individual which accounts for uncertainty due to low sequencing depth^142^. Genotype likelihoods were estimated using the Samtools GL model, and the major and minor allele frequencies were estimated with the EM algorithm^143^. Only sites with significant evidence for polymorphism (*P* < 1 × 10^−6^) and a minor allele frequency (MAF ≥ 0.05) were included. Read quality filters were set using minimum mapping (minMapQ ≥ 30) and base qualities (minQ ≥ 20). Only sites present in at least 80% of the samples were kept. We excluded the sex chromosome (chromosome 19) to avoid potential sex biases in the downstream analysis.

The genetic relationships among all individuals and populations was explored with pairwise genetic distances estimates with ngsDist v1.0.8^144^ with branch support based on bootstrapping 500 replicates with 500 SNP blocks and pairwise deletion. The phylogenetic tree was inferred with FastME v2.1.5 using a BME iterative taxon addition method with SPR tree refinement^145^. Support values were mapped using RAxML-NG v0.9.0^146^. The final bootstrapped tree was visualized and plotted using the online software iTOL v5^147^.

### Whole genome sequencing

Using the phylogenetic tree as a reference (Fig. 1A), we strategically targeted four divergent geothermal/ambient ecotype pairs for whole-genome sequencing. The four geothermal/ambient ecotype pairs were subsampled based on variation in their demography (sympatry vs. allopatry) as well as their degree of phylogenetic divergence. Grettislaug (GTS) and Garðsvatn (GAR) are an evolutionary old allopatric pair of geothermal and ambient ecotypes, respectively. Mývatn (MYV) represented a pair of evolutionary old sympatric geothermal and ambient ecotypes. Áshildarholtsvatn (ASHN) represented a pair of evolutionary young (diverged within the last 70 years) sympatric geothermal and ambient ecotypes. Lastly, Sauðárkrókur (SKR) represented a pair of evolutionary young (diverged within the last 70 years) allopatric geothermal and ambient ecotypes. The four geothermal and ambient ecotype pairs were sampled to identify genomic patterns relating to thermal habitat divergence regardless of demographic history and age of divergence.

We sampled individuals across the four geothermal/ambient pairs for whole-genome sequencing (*n* = 109). Each geothermal and ambient ecotype had roughly fourteen individuals (GTS geothermal = 14; GAR ambient = 14; MYV geothermal = 12; MYV ambient = 13; ASHN geothermal = 14; ASHN ambient = 14; SKR geothermal = 14; SKR ambient = 14). Genomic DNA was extracted from each individual from fin clips using the Quigene DNA extraction kit and were run on the Novoseq 6000 at the Center for Genomic Research at the University of Liverpool (SKR, GTS, GAR) and a HiSeqX at the University of Edinburgh (MYV, ASHN). Reads were mapped to the stickleback genome^138^ using BWA-MEM(version 0.7.5a)^139^ with the default parameters. Alignments were then filtered to remove reads with a mapping quality lower than 10, which equates to a 90% chance that the read was derived from another genomic location. Read duplicated were identified and filtered to retain a single representative using the Picard “MarkDuplicates” tool, version 1.85 (http://picard.sourceforge.net/). Variant detection was performed using the GATK HaplotypeCaller tool (v4.3.0.0)^148,149^. Variants were then filtered using the GATK VariantFiltration tool (McKenna et al., 2010) following assessments of GATK recommended cut-offs. i.e., SNPS with either QD < 2, QUAL < 30, FS > 60, MQ < 40, SOR > 3, MQRankSum < −12.5 or ReadPosRankSum < −8 were flagged “, and indels with QD < 2, QUAL < 30, FS > 200, ReadPosRankSum < −20 were flagged. Single nucleotide polymorphisms (SNP) were filtered for minor allele frequencies less than 0.05 using vcftools^150^, and pruned for linkage disequilibrium using the –indep 50 5 2 flag in plink 1.9^151,152^. After the filtering steps, we were left with 173,485 biallelic SNPs which were polymorphic in at least a single population.

### Population genetic structure based on whole genomes

We characterized fine-scale population genetic structure and admixture across the four geothermal/ambient ecotype pairs. First, we determined the genetic relationship among all populations using a principal component analysis (PCA). The PCA was performed using pcadapt^153^ which is based on Mahalanobis distance and a MAF cut-off of 0.01. Second, we assessed the amount of admixture and distinct genetic structure between the geothermal and ambient morphs from all populations using ADMIXTURE^154^. We tested K values from 1:10 with a cross-validation procedure and the default parameters of ADMIXTURE. The optimal K value was determined as having the lowest cross-validation error. Third, we calculated Fst between all geothermal and ambient ecotype pairs to estimate the degree of overall genomic differentiation of the thermal ecotypes using the *stamppFST* function from the StAMPP package^155^.

### Local adaptation to geothermal habitats

We identified regions of the genome that were potentially under selection and differentiated geothermal and ambient ecotypes. We calculated per-locus Fst between geothermal and ambient ecotypes using plink (version 1.9)^151,152^. We then identified Fst outlier loci as any locus within the top 0.5% of the Fst distribution. In order to keep Fst outliers that are biologically meaningful, we further filtered the outlier loci by only keeping loci that were greater than the average Fst of the outlier distribution for each geothermal/ambient ecotype pair. We then compared the degree of overlap of Fst outliers between geothermal/ambient ecotype pairs and performed a Fishers exact test. We then identified the number of Fst outliers that corresponded to gene (coding) regions for each geothermal/ambient ecotype pair. Lastly, we assessed the degree of overlap in outlier genes between each ecotype pair using a Fishers exact test and performed a gene ontology (GO) analysis for each ecotype pair using Metascape^156^. We then tested whether the degree of shared outliers and genes (measured by the Sørensen–Dice distance) were predicted by genome-wide divergence (FST), as a proxy for population age, using multiple regression on distance matrices with 1,000,000 permutations (*ecodist* package^157^).

### Parallel adaptation to geothermal habitats

We identified divergent regions of the genome which were common to all geothermal/ambient ecotype pairs. To minimize the amount of noise across the genome, we performed a sliding window analysis using 25 Kilobase (Kb) windows with an overlap between windows of 1 Kb using the R package windowscanr (github.com/tavareshugo/WindowScanR). We then filtered the dataset to include windows containing at least three SNPs within the window and defined Fst outliers as being in the top 0.5% of the Fst distribution and have a Fst value greater than the average Fst from the outlier distribution.

We then identified regions of the genome diverging in parallel between all geothermal and ambient adapted ecotypes. We conducted a scan to determine parallel changes in allele frequencies across all geothermal and ambient adapted ecotypes using an allele frequency vector analysis of parallel evolutionary responses, implemented using the R package AF-vapeR^158^. Each chromosome was split into 50 SNP windows, and a matrix of allele frequencies were calculated for each window. Each cell within the matrix represented a change in allele frequency between geothermal and ambient ecotypes for a given SNP. An allele frequency vector was calculated for all SNPs within a given window, and then the allele frequency vector was normalised so that all population pairs had equivalent variance. Correlation coefficients were then calculated between the normalised allele frequency vectors, and eigenvector decomposition was performed on the correlation coefficients for a given 50 SNP window. The changes in allele frequency vectors were then compared to a null distribution made up of randomized allele frequency changes across the populations. Full parallelism of allele frequencies were indicated as elevated values on the first eigenvector, while multi-parallelism and divergent allele frequencies were indicated as elevated values on eigenvectors two and three.

Any chromosome showing significant parallel or multi-parallel changes in allele frequencies among geothermal and ambient adapted morphs were assessed for selective sweeps. We assessed regions of the genome that appeared to be diverging in parallel (or multi-parallel) trajectories for selective sweeps using the R package rehh^159^. We used the rehh package as it allows for cross-population comparisons of extended homozygosity haplotypes based on the commonality of allele frequencies. We used the *scan_hh* function to calculate major and minor allele frequencies and the integrated haplotype homozygosity score (iHH) for each marker along a chromosome. As we did not know which markers were derived or ancestral we specified that the markers were not polarized allowing for the use of allele frequencies. We then used the function *ies2xpehh* to calculate extended homozygosity haplotypes between geothermal and ambient morphs based on allele frequencies; a Bonferroni correction was applied. Generally, values above 0 were associated with geothermal ecotypes and values below 0 were associated with ambient ecotypes.

Regions of the genome which showed parallel or multi-parallel changes in allele frequencies were also investigated to be associated with putative structural variants such as inversions. First, we performed a PCA, using the glPca function in adegenet^160^, with k-means clustering, using the kmeans function, on the entire region that is diverging in parallel to identify the inversion karyotypes. As local PCA methods (i.e., sliding window analyses) have been shown to reliably detect structural variants, such as inversions, from short read sequencing data^93,94^. We used the lostruct package^93^ to identify outliers in population structure along chromosomes containing significant and (multi) parallel changes in allele frequencies. We used a window size of 50 SNPs and used the eigen_windows function to perform the local PCA along the chromosome containing the region of interest. We then used the pc_dist function to calculate a distance matrix between all windows in the previous analysis. Multidimensional scaling analysis, using the cmdscale function, was applied to the distance matrix to identify which PCA windows were population structure outliers.

Lastly, we identified all genes within regions that showed parallel or multi-parallel changes in allele frequencies and performed a gene ontology analysis. Gene ontology analysis can be used to identify terms that are associated with the input genes as well as biological processes. We enrichR^161^ to identify significant gene ontology terms associated with our genes of interest as enrichR performs better with a smaller set of candidate genes compared to Metascape^162^. For both gene ontology analyses, we converted the threespine stickleback gene ids to homologous gene ids in the Zebrafish genome (*Dania rerio*), and the threespine stickleback genome annotation was not available. For the enrichR gene ontology analysis, we used the diabetes, Biocarta, enrichr, GO biological process, and KEGG databases.

### Expression of genes in the MAPK pathway

To assess the divergence and plasticity of genes expressed in the *mapk* pathway, we conducted common garden experiments combined with RNA-seq to identify genes in the *mapk* pathway with divergent and plastic expression between geothermal and ambient ecotypes. The first common garden experiment tested for common gene expression changes between ecotypes and temperatures across population pairs. A second common garden experiment was used to test the effects of ecotype hybridization on the expression of *mapk* genes identified in the first common garden experiment. Together, these experiments tested the functional significance of MAPK genes between thermal ecotypes, temperature, and hybridization.

The first common garden experiment used F1 geothermal and ambient ecotypes. To start, F1 offspring were made for geothermal and ambient ecotypes from ASHN, MYV, and SKR populations. For each population pair, males displaying mating colouration were euthanized by benzocaine overdose, with the testes were extracted, and placed in a sperm storage solution in a 1.5 mL Eppendorf tube on ice^163^. Eggs were stripped from gravid females of each thermal ecotype and fertilized with sperm from a male of the corresponding ecotype to make pure geothermal-geothermal or ambient-ambient crosses for each ecotype pair^38^. Each clutch was equally divided between temperature treatments of 12 °C and 18 °C until maturity (~10 months of age) and euthanized by benzocaine overdose for collection of tissues. The livers were dissected out, placed in room temperature RNA later for 24 h, and then put into −80°C for storage. RNA was extracted from approximately 20 mg of liver tissues using Qiagen RNeasy extraction kits using 50% ethanol for washes as recommended for fatty tissue. In total, RNA was extracted from 163 individuals between three populations, two thermal ecotypes, and two rearing temperatures (Table S4). The preparation of cDNA libraries and RNA-seq was performed at the Center for Genomic Resources at the University of Liverpool. Library preparation was performed using Qiagens 3’ UPX transcriptome kit to generation pools of 24 samples, which were then sequenced using the Novaseq platform.

We then assessed divergence and plasticity in expression of *mapk* genes. The Fastq files were de-multiplexed using *demultiplex* function in the Qiagen CLC workbench (version 23.0.4) following the QIAseq sample analysis workflow. Sequences were trimmed for the presence of Illumina adapters using *Cutadapt* version 1.2.1^164^ and further trimmed using *Sickle* version 1.200 (https://github.com/najoshi/sickle). Read quality was checked using *FastQC* (https://github.com/s-andrews/FastQC) and *MultiQC*^165^. RNA-seq reads were aligned to version 5 of the threespine stickleback genome^166^ using the *Hisat2* aligner (version 2.2.0)^167^, samples with greater than 10 million aligned reads were retained, and read counts were generated using *Htsqcount2*^168^. Low-expressed genes were filtered using the *filterbyExpr* function, and read counts were normalized by calculating scaling factors using the *calcNormFactors* function in the *LIMMA* R package^169^. We then used a generalized linear model (GLM) with a non-linear minimization optimizer to model gene expression changes across 14,566 genes: population * ecotype * temperature + (1/family effects) using the *lme4* package^170^. The model fits an association of expression changes per gene while accounting for expression of all other genes with each fixed effect while family effects were fitted as a random effect (https://github.com/PhDMattyB/Bethany_gene_expression). We then filtered the genes with a significant (*p*-value < 0.05) ecotype-temperature interaction, performed a Benjamini-Hochberg correction to account for false discovery rates, and filtered the significant genes (*p*-value < 0.05) for all known *mapk* genes available from the KEGG pathway^171,172^. Lastly, we did a gene ontology (GO) analysis on all genes identified using Metascape^156^.

sTo assess the effects of hybridization on expression of *mapk* pathway genes relevant to our genomic findings, we conducted a second common garden experiment on F1 individuals form the SKR population. F1 crosses were made in the same way as the previous common garden experiment using males and females from the SKR ecotype pair however, we made geothermal-ambient hybrids with the same ambient female and geothermal male for two families. The geothermal, ambient, and hybrid clutches were divided equally between 12˚ °C and 18 °C temperature treatments until maturity. At maturity, the F1 offspring were euthanized by benzocaine overdose and the brains were dissected out, placed in room temperature RNA later for 24 hours, and then frozen at −80°C for storage. A total of 48 individuals were sampled (Table S4) with 8 individuals sampled per ecotype per temperature. RNA was extracted from approximately 20 mg of brain tissue using Qiagen RNeasy lipid tissue extraction kits. The preparation of cDNA libraries and RNA-seq was performed at the Center for Genomic Resources at the University of Liverpool. Library preparation was performed using the NEBNExt Poly(A) mRNA magnetic isolation modules and NEBNext Ultra RNA library prep kit for Illumina, which were then sequenced using the Novaseq platform with an average read length of 150 base pairs and 20 million reads per sample.

We assessed the effects of hybridization on divergence and plasticity of transgressive expression of MAPK signalling pathway genes. The Fastq files were de-multiplexed using *demultiplex* function in the Qiagen CLC workbench (version 23.0.4) following the QIAseq sample analysis workflow. Sequences were trimmed for the presence of Illumina adapters using *Cutadapt* version 1.2.1^164^ and further trimmed using *Sickle* version 1.200 (https://github.com/najoshi/sickle). Read quality was checked using *FastQC* (https://github.com/s-andrews/FastQC) and *MultiQC*^165^. RNA-seq reads were aligned to version 5 of the threespine stickleback genome^166^ using the *Hisat2* aligner (version 2.2.0)^167^, samples with greater than 20 million aligned reads were retained, and read counts were generated using *Htsqcount2*^168^. Low-expressed genes were filtered using the *filterbyExpr* function, and read counts were normalized by calculating scaling factors using the *calcNormFactors* function in the *LIMMA* R package^169^. We used LIMMA to model changes in gene expression between pure strain geothermal and ambient ecotypes and hybrids at each rearing temperature using a contrast matrix for 13452 total genes normalized using a voom transformation to control for expression variance. For each of the four contrasts (geothermal-hybrid at 12°C; geothermal-hybrid at 18°C; ambient-hybrid at 12°C; and ambient-hybrid at 18°C), we identified genes as being transgressively expressed, over- or under-dominant expression, as in ref. ^81^. We then performed a Bonferroni correction to account for false discovery rates, filtered the genes for significance (*p*-value < 0.05), and filtered the gene set for all known MAPK genes available from the KEGG pathway^171^. We identified overlap in MAPK genes between all four LIMMA contrasts as well as overlap in genes previously identified as being divergent and plastic in response to temperature in the previous common garden experiment.

### Appetite, mass, and fat content phenotypes

To assess phenotypic variation relevant to our genomic findings we conducted appetite feeding trials, food restriction experiments, and assessments of body fat in fish from both geothermal and ambient habitats. For appetite trials we used lab bred F2 generation fish from ASHN, MYV, and SKR. We reared fish to a mature stage and following a fast of 24 h we fed individuals (*n* = 18 per ecotype per lake) standard aquaculture pellets (EWOS, Weybridge, Surrey, Start 040 inc Boosterfeed, pellet size 0.9 mm) to satiation. Satiation was defined as the point at which fish stopped feeding or were unable to consume more food. We counted the number of pellets that each individual ate as well as the time take to eat to the point of satiation. We used a type II ANOVA, using the *aov* function in the stats package, to test for the effects of population, ecotype, and their interaction on the number of pellets eaten to satiation as well as the time to satiation (model 1: Number of pellets eaten ~ Population * Ecotype; model 2: Time to satiation ~ Population * Ecotype).

Our tests for differences in weight loss under a restricted diet were conducted on wild fish from ASHN and MYV ecotype pairs. We collected between 17 and 26 individuals per ecotype per lake (41 ambient and 50 geothermal individuals total) in the summer of 2017. We then measured the initial total mass of each fish and tagged them with visible implant elastomers to enable individual identification before subjecting them to a reduced food intake for 30 days^112^. After the 30 day period we weighed each individual to calculate the amount of weight lost by each individual. We tested for differences in the amount of mass lost between geothermal and ambient ecotypes due to the treatment with a repeated measures ANOVA using the *anova_test* function in the *rstatix* package (https://github.com/kassambara/rstatix).

To test for differences in body lipid content between wild ecotypes caught in summer and winter of 2017 from five populations in Iceland. We collected between 18 and 20 individuals per ecotype in the summer and between 20 and 32 individuals per ecotype in the winter from ASHN, SKR, GTS, GAR, and STN (a location in the north of Iceland with geothermal stickleback). Collected stickleback were euthanized by phenoxyethanol overdose and fixed in 10% formalin in the field. We used diethyl ether to extract fat from each individual to assess fat content across different seasons following the protocol outlined in Auer et al. (2016). Individuals were dehydrated and weighed before being submerged in diethyl ether within a 50 ml falcon tube for a period of 7 days to dissolve fat. Following this, individuals were once again dehydrated and weighted to obtain a difference in weight. We then used an ANOVA to model variation in lipid content due to population, ecotype, and season (Lipid content ~ Population * Ecotype * Season) using the *aov* function in the *stats* package.

## Supplementary information

Supplementary Information

Peer Review file

## Source data

Source Data

## Data Availability

Data used to generate the results of the manuscript is available on FigShare: https://doi.org/10.6084/m9.figshare.29075546, https://doi.org/10.6084/m9.figshare.31972392, https://doi.org/10.6084/m9.figshare.31972485. Raw sequencing data have been deposited on NCBI in BioProject PRJNA1467975. Source data are provided with this paper.

## Code availability

All code used to generate the results of the manuscript is available on GitHub: https://github.com/PhDMattyB/Nature_comms.
