# Supplementary material for: Parallel adaptation to geothermally-warmed habitats due to common structural variation and functional developmental pathways
Source: Nat Commun. 2026 Jul 22;17:10103. doi: 10.1038/s41467-026-75785-0 (PMC13598157; doi:10.1038/s41467-026-75785-0)
Supplement: Supplementary file 1 [file 41467_2026_75785_MOESM1_ESM.pdf]

Supplementary Information

Supplementary tables and figures

Table S1. Sites (n = 25) sampled for threespine stickleback for the phylogeographic analysis.

Site name	Site code	Coordinates
Grettislaug	GTS	65.88224 -19.73661
Reykir	RKRW	65.47086 -19.358078
Gardsvatn	GAR	65.73074 -19.462626
Holmavatn	HGRNS	65.675167 -19.486183
Saudarkrokur Cold	SKRC	65.732191 -19.618574
Saudarkrokur Warm	SKRW	65.732260 -19.618937
Steinsstadir	STNST	65.469154 -19.358097
Myvatn Warm	MYVTNW	65.633991 -16.923241
Myvatn Cold	MYVTNC	65.630196 -16.991621
Kleifarvatn Cold	KLFC	63.93811 -21.94195
Thingvallavatn Warm	THNGW	64.15059 -21.200521
Litlaa	LITA	66.076947 -16.684749
Litlaa Pond	LITP	66.082295 -16.681183
Skjalftavatn	SKAL	66.077446 -16.676031
Reykholt Warm	RKLTW	64.6618 -21.374348
Reykholt Cold	RKLTC	64.67892 -21.2506
Ashildarholtsvatn Warm	ASHNW	65.72516 -19.600725
Ashildarholtsvatn Cold	ASHNC	65.724976 -19.601784
Reykir River Cold Site	RKRC	65.469936 -19.360365
Behind barn on farm	BARN	65.4764 -19.3697
Ljosaland	NH	65.4761 -19.3748
Thingvallavatn Cold	THNGC	64.186742 -21.094086
Opnur Warmer end	OPNUR	63.978877 -21.17661
Marine	HERD	Unknown
Marine	NYPS	Unknown

Table S2. Overlap between Fst outlier loci and genes associated with Fst outlier loci across geothermal and ambient ecotype pairs of Icelandic threespine stickleback. The number of overlapping Fst outlier loci are shown below the diagonal while the number of overlapping genes associated with Fst outlier loci are show above the diagonal.

	**ASHN**	**MYV**	**SKR**	**GTS-GAR**
**ASHN**	-	0	1	1
**MYV**	0	-	4	2
**SKR**	1	1	-	3
**GTS-GAR**	1	1	3	-

Table S3. Networks of genes which have proteins that form physical interactions for each geothermal-ambient ecotype pair across five populations of Icelandic threespine stickleback. Gene lists were formed for each geothermal-ambient ecotype pair based on their physical location to Fst outlier loci.

Population	Network	Gene list
**ASHN**	Network 1	cacnalaa
		nalcn
		fgf13b
	Network 2	prkceb
		map2k5
		map3k22
**MYV**	Network 1	mapk1
		plxnaa2
		prprfb
		prprua
**SKR**	Network 1	ptprea
		ptprfb
		epha4a
		itgb5
		cdh13
		fgfr2
		mapk13

		mapk1
		epha6
**GTS-GAR**	Network 1	ubea2wa
		ppp1r42
		mybh1
		myb
		lrrk1
		tra2a
		sgk3
	Network 2	mtrr
		ppp3cb
		trpa1b

Table S4. Number of samples used for RNA-seq on liver tissue from a common garden experiment.

**Common garden experiment 1**			
**Population**	**Ecotype**	**Temperature**	**Number of samples**
ASHN	Ambient	12	13
ASHN	Ambient	18	14
ASHN	Geothermal	12	17
ASHN	Geothermal	18	15
MYV	Ambient	12	16
MYV	Ambient	18	15
MYV	Geothermal	12	17
MYV	Geothermal	18	16
SKR	Ambient	12	7
SKR	Ambient	18	11
SKR	Geothermal	12	11
SKR	Geothermal	18	11
**Common garden experiment 2**			
**Population**	**Ecotype**	**Temperature**	**Number of samples**
SKR	Ambient	12	8
SKR	Ambient	18	8
SKR	Hybrid	12	8
SKR	Hybrid	18	8
SKR	Geothermal	12	8
SKR	Geothermal	18	8

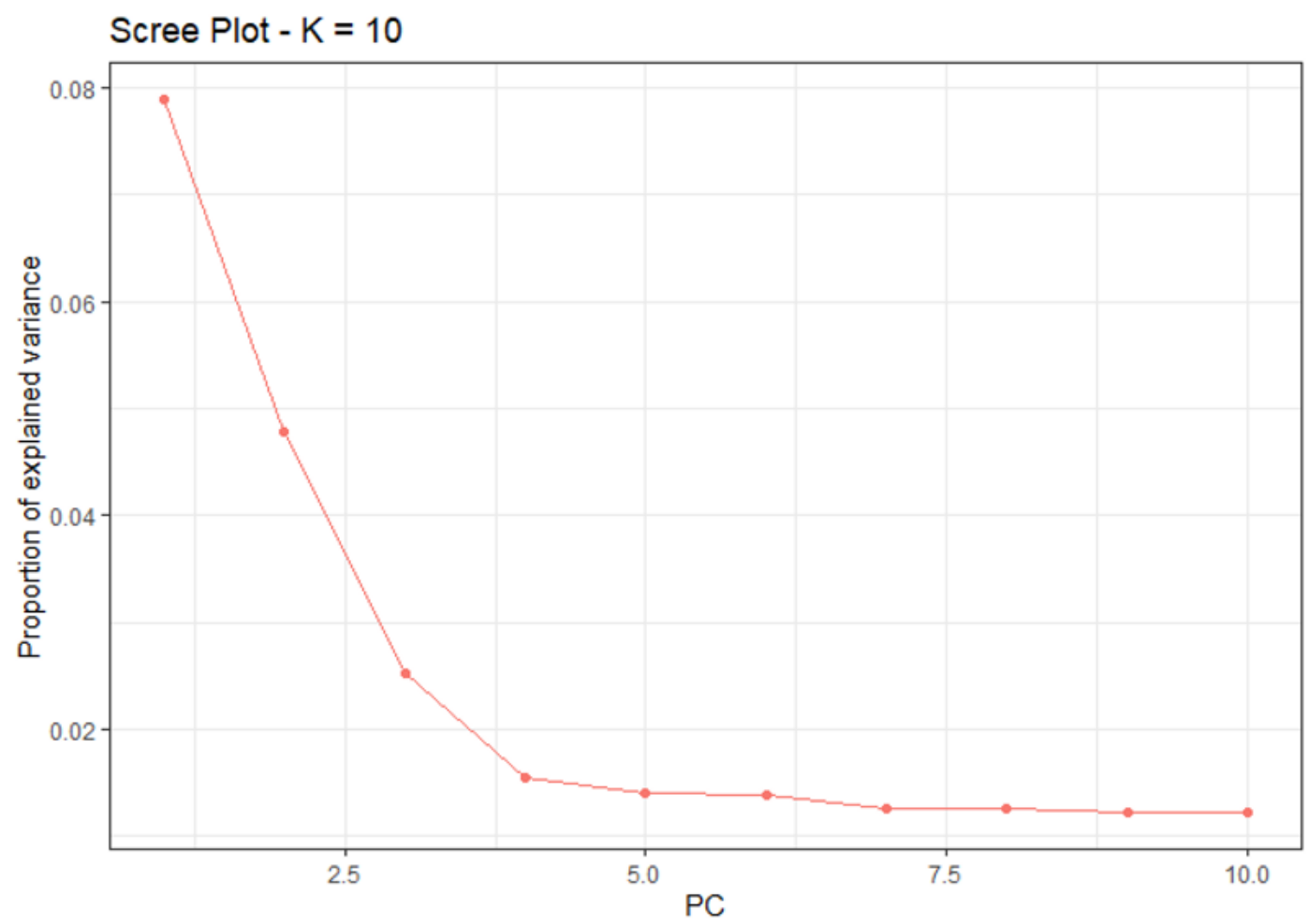

Figure S1. Evaluation of population genomic structure. Scree plot to determine the number of principal components to retain representing the number of populations. The number of principal components to retain is indicated by the elbow and K=3.

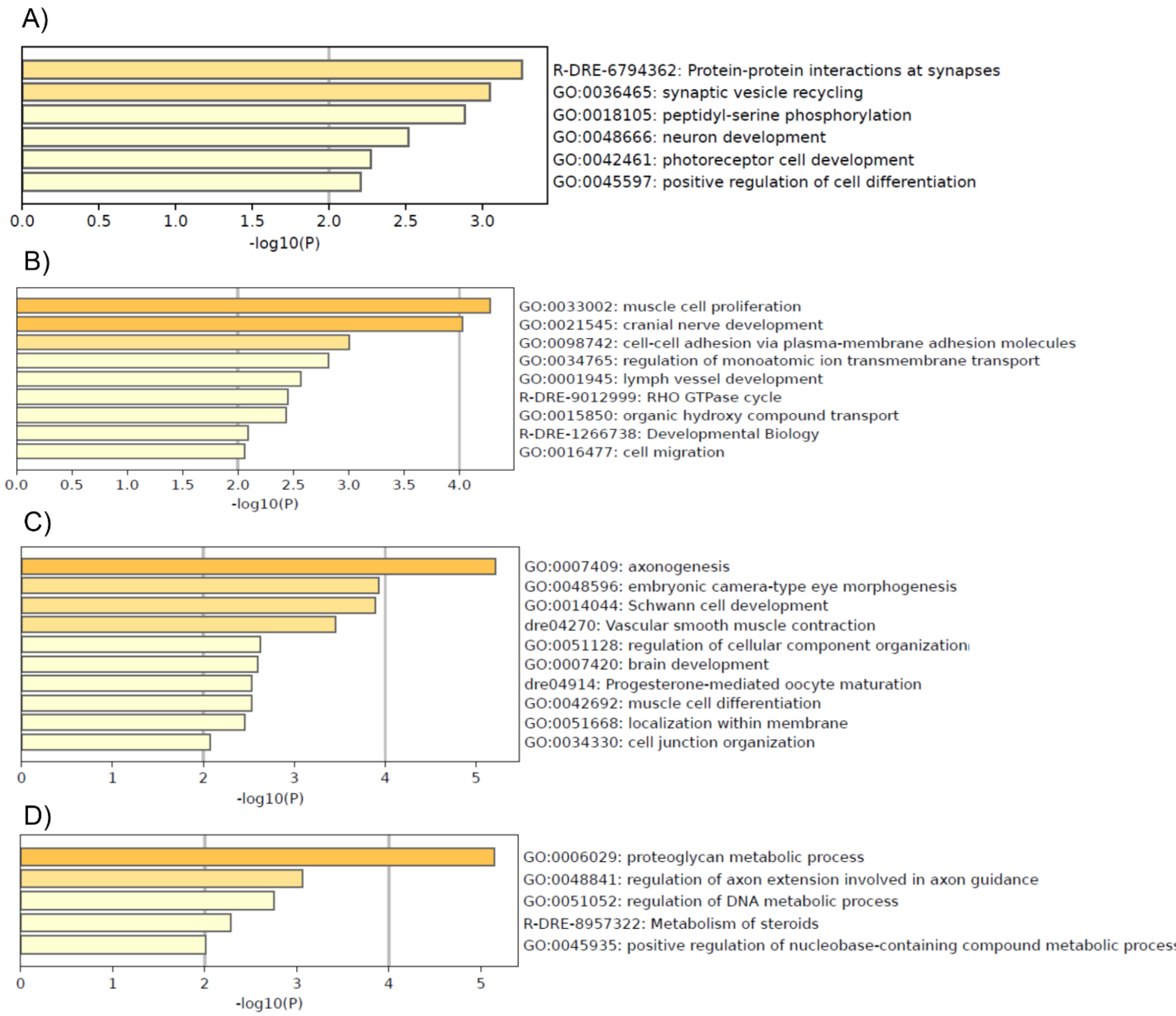

Figure S2. Gene ontology enriched terms (molecular function) from differentiated genes between geothermal and ambient ecotypes of Icelandic threespine stickleback. A) Áshildarholtsvatn geothermal and ambient ecotypes. B) Mývatn geothermal and ambient ecotypes. C) Sauðárkrókur geothermal and ambient ecotypes. D) Geothermal ecotypes from Grettislaug and ambient ecotype from Garðsvatn.

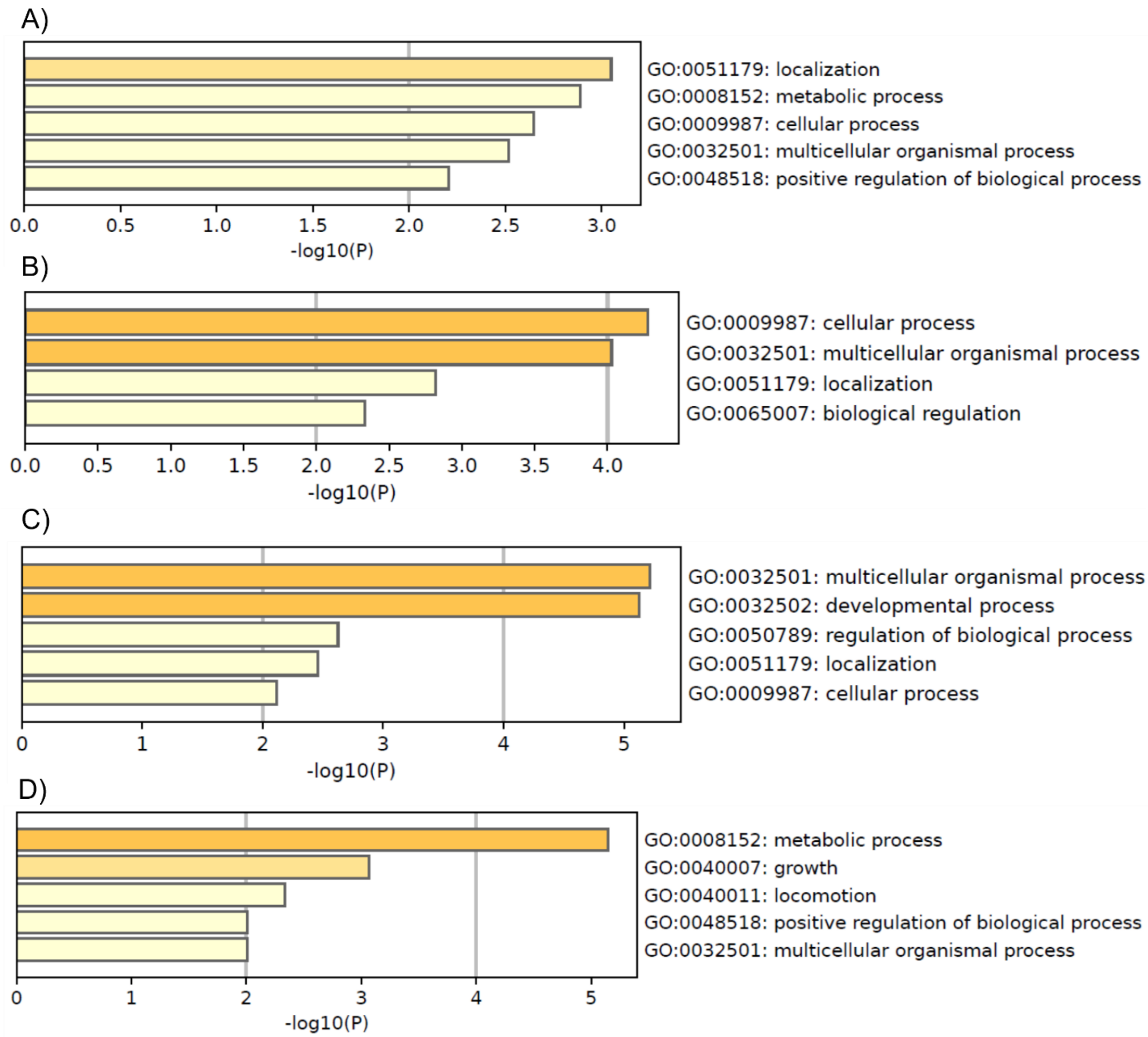

Figure S3. Gene ontology enriched terms (biological process) from differentiated genes between geothermal and ambient ecotypes of Icelandic threespine stickleback. A) Áshildarholtsvatn geothermal and ambient ecotypes. B) Mývatn geothermal and ambient ecotypes. C) Sauðárkrókur geothermal and ambient ecotypes. D) Geothermal ecotypes from Grettislaug and ambient ecotype from Garðsvatn.

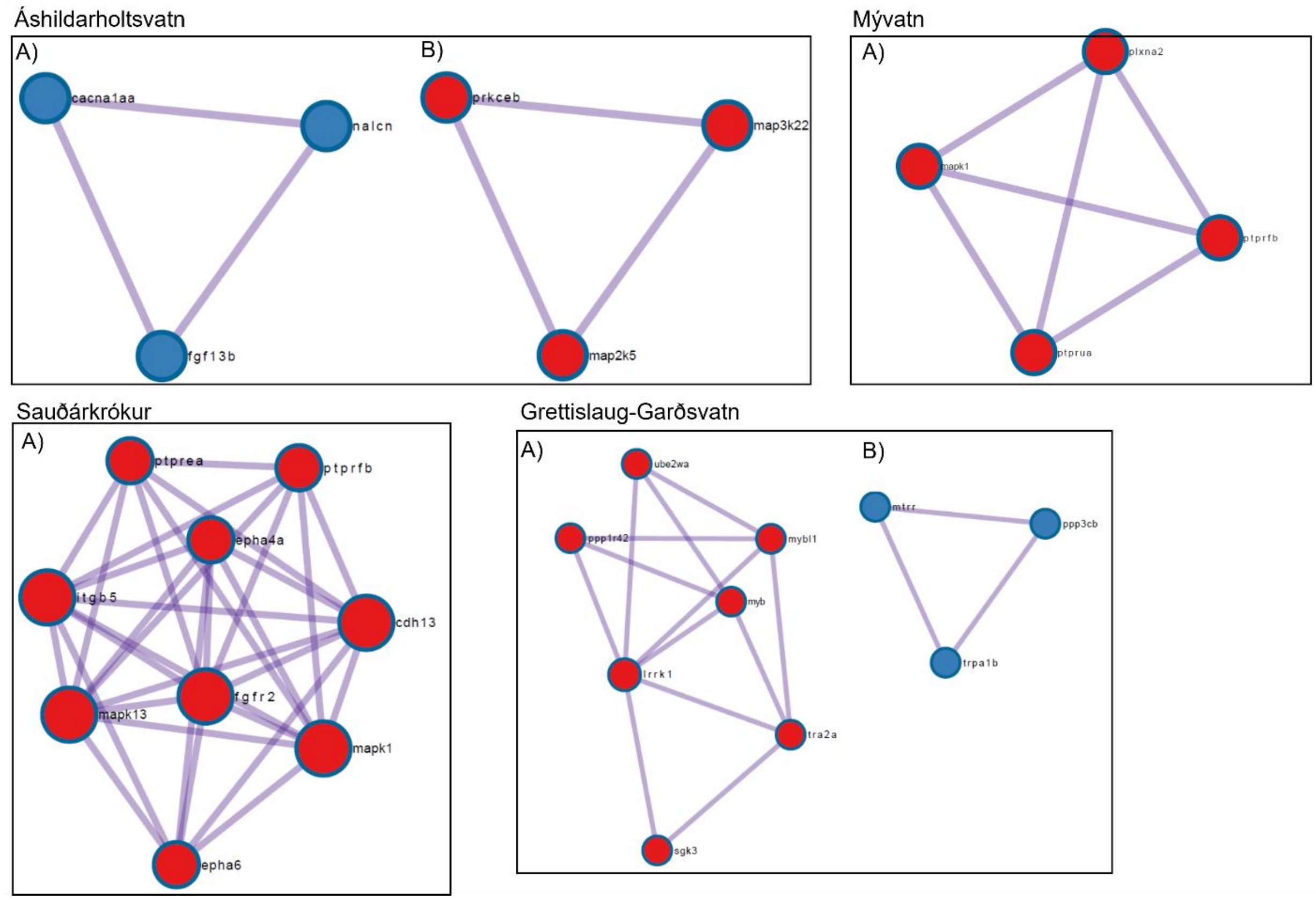

Figure S4. Protein-protein interaction enrichment analysis from differentiated genes between geothermal and ambient ecotypes of Icelandic threespine stickleback. A) Áshildarholtsvatn geothermal and ambient ecotypes. B) Mývatn geothermal and ambient ecotypes. C) Sauðárkrókur geothermal and ambient ecotypes. D) Geothermal ecotypes from Grettislaug and ambient ecotype from Garðsvatn.

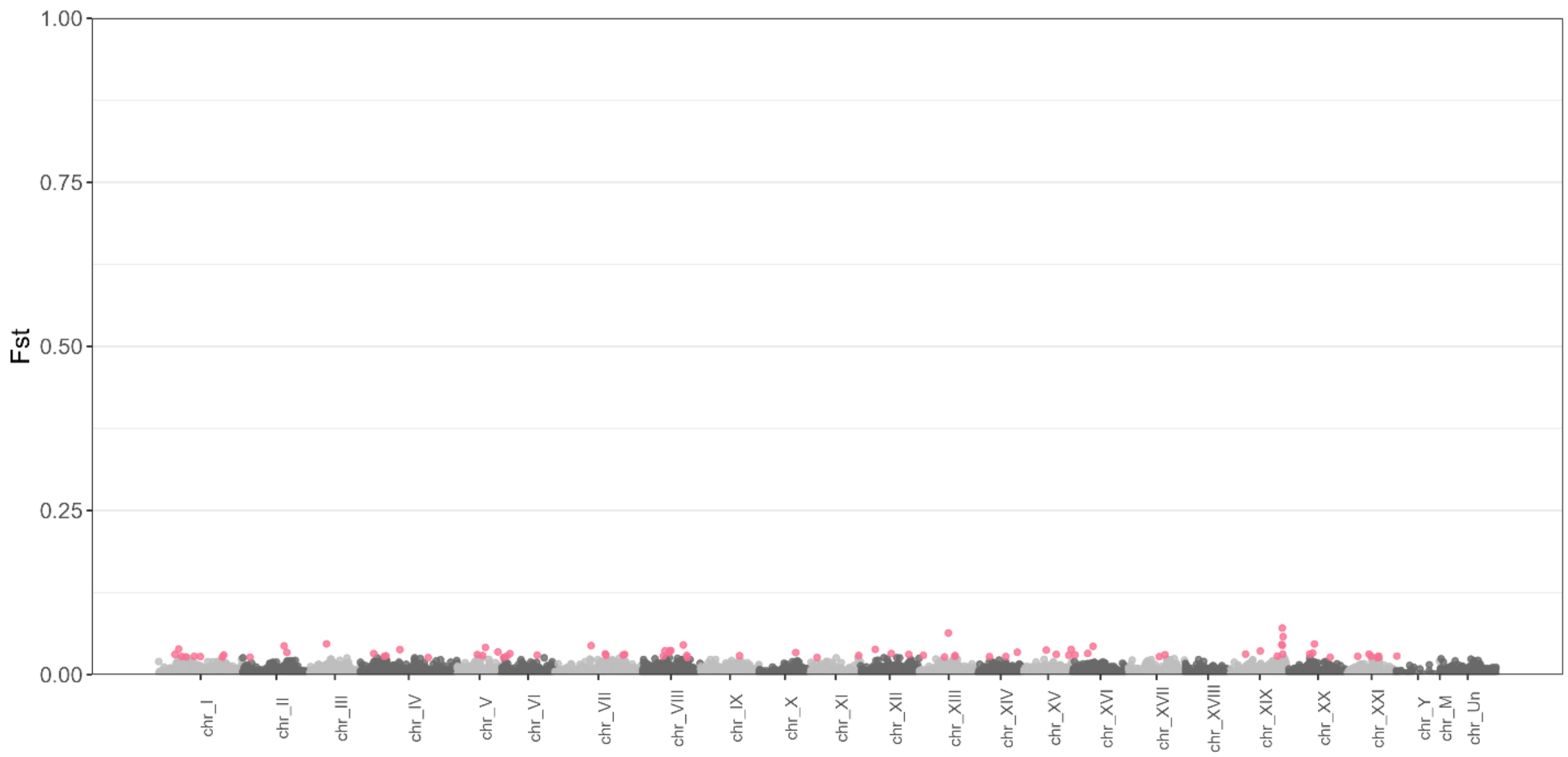

Figure S5. Fst outliers from a sliding window analysis (25Kb windows) that differentiate all geothermal and ambient ecotypes, regardless of population of origin. The Fst outlier windows are shown in pink and the neutral windows are shown in shades of grey. Fst outliers were in the top 5% of the overall Fst distribution.

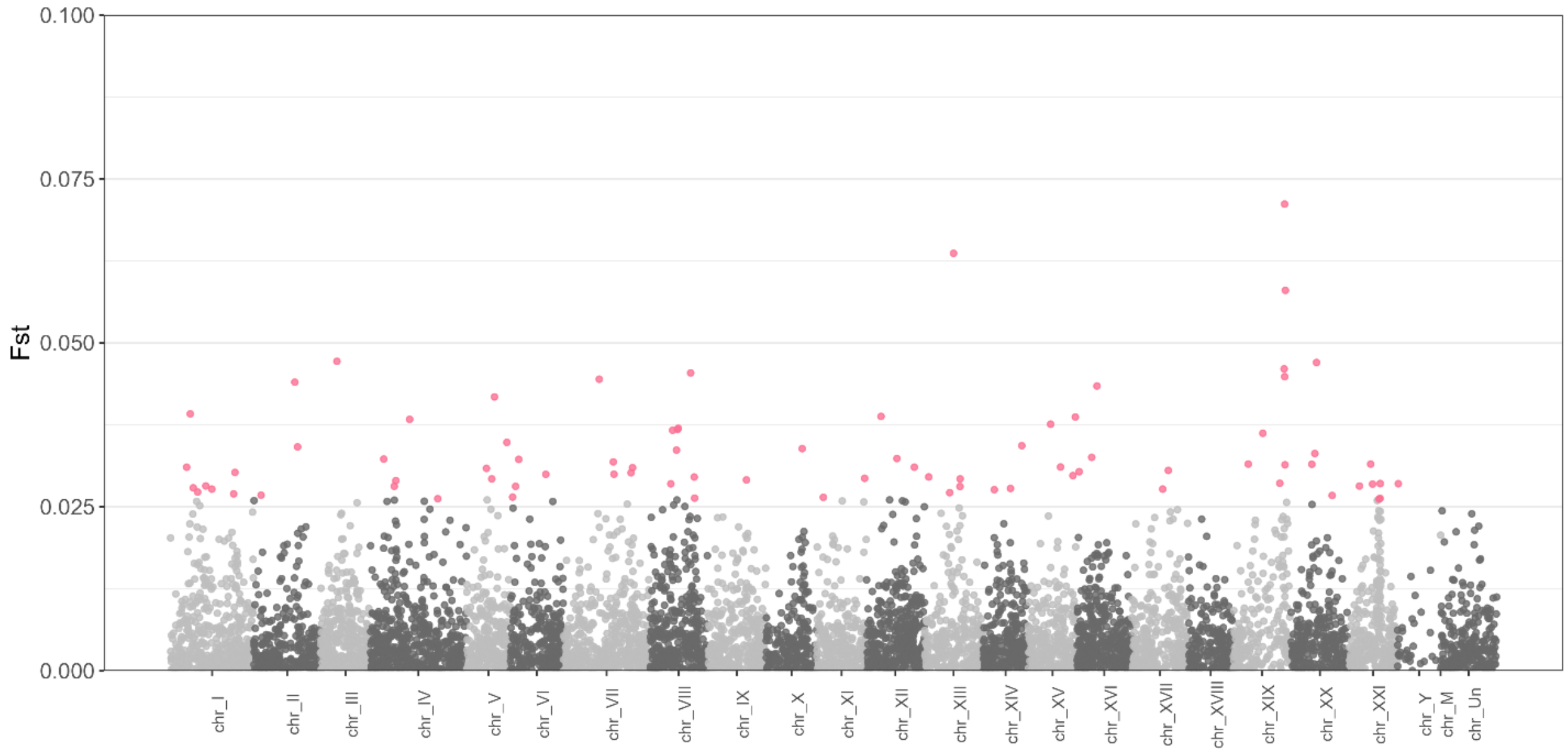

Figure S6. Per-locus Fst outliers differentiating all geothermal and ambient ecotypes, regardless of population of origin. The Fst outliers are shown in pink and neutral loci are shown in shades of grey. Fst outliers are shown as the top 5% of the Fst distribution.

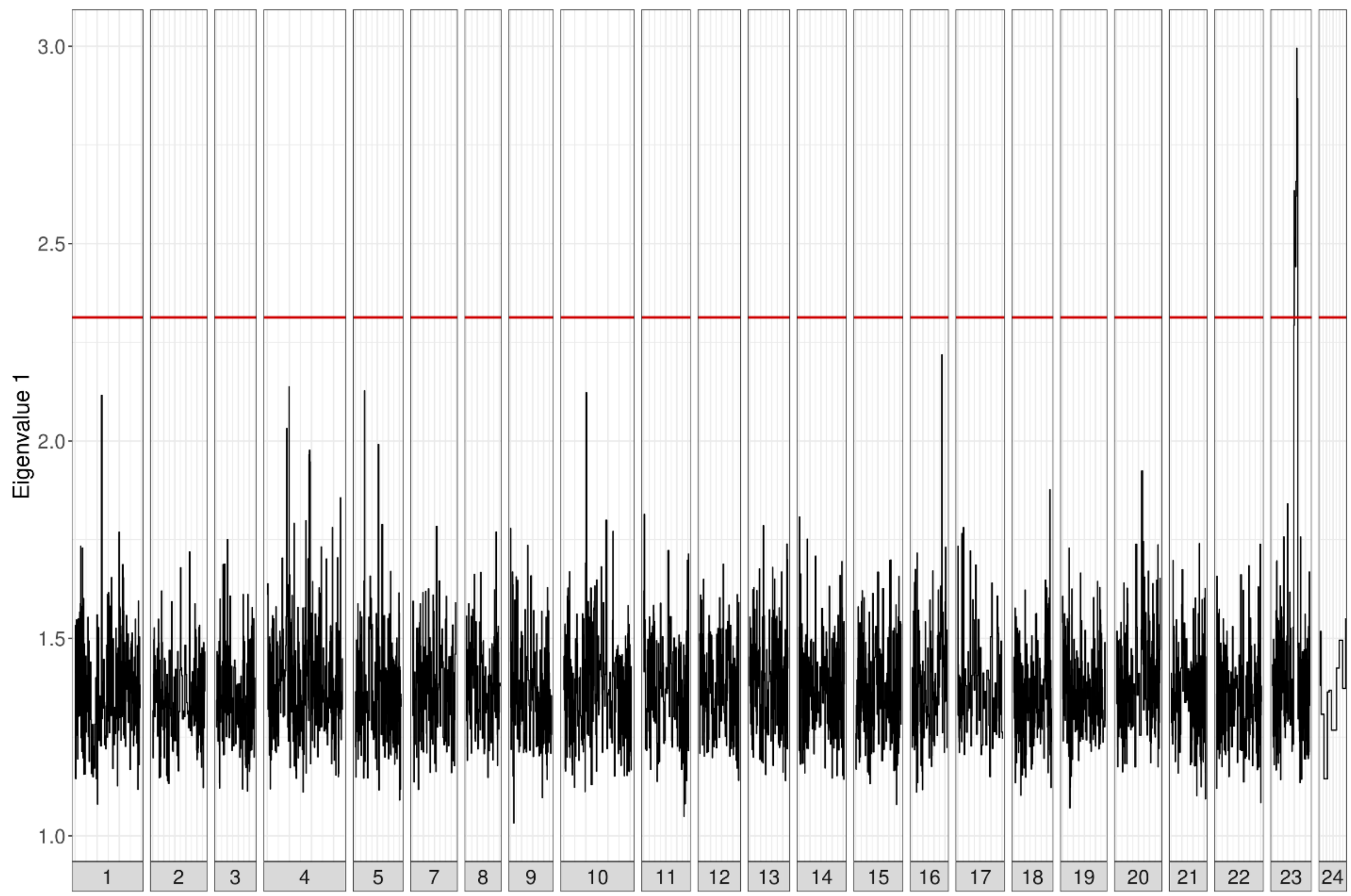

Figure S7. Eigenvalues of sliding window analysis (50 SNP windows) for the parallel differentiation of allele frequencies (AFvapeR) between geothermal and ambient ecotype pairs. The red line indicates the significance cut-off. Chromosome 23 (chromosome XXI) is the only chromosome with significant windows that differentiate geothermal and ambient ecotypes in parallel.

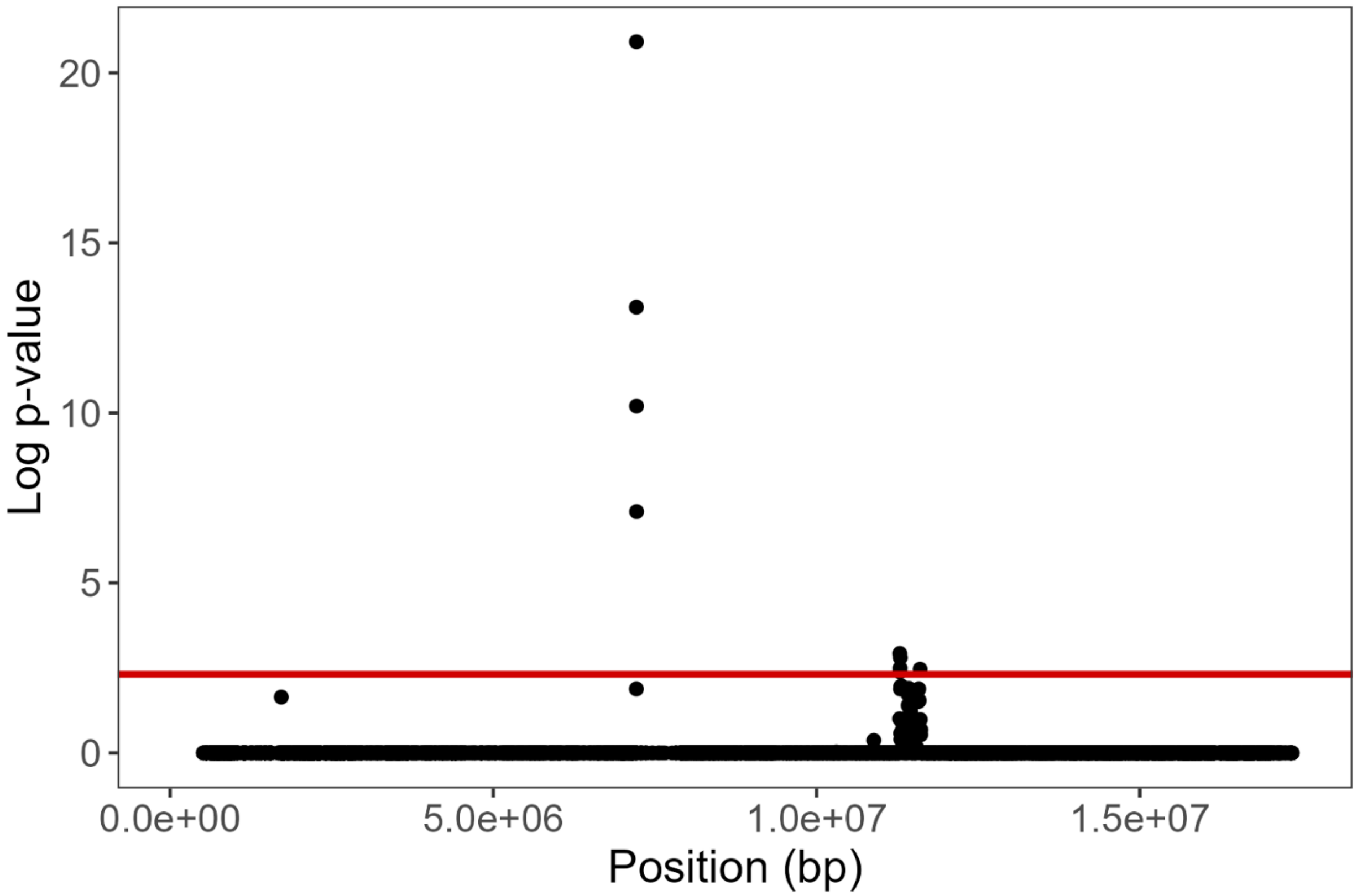

Figure S8. P-values of sliding window analysis (50 SNP windows) of the parallel differentiation of allele frequencies (AFvapeR) between geothermal and ambient ecotype pairs. The p-values are shown for the region of parallel differentiation on chromosome XXI. The red line indicates the significance cut-off.

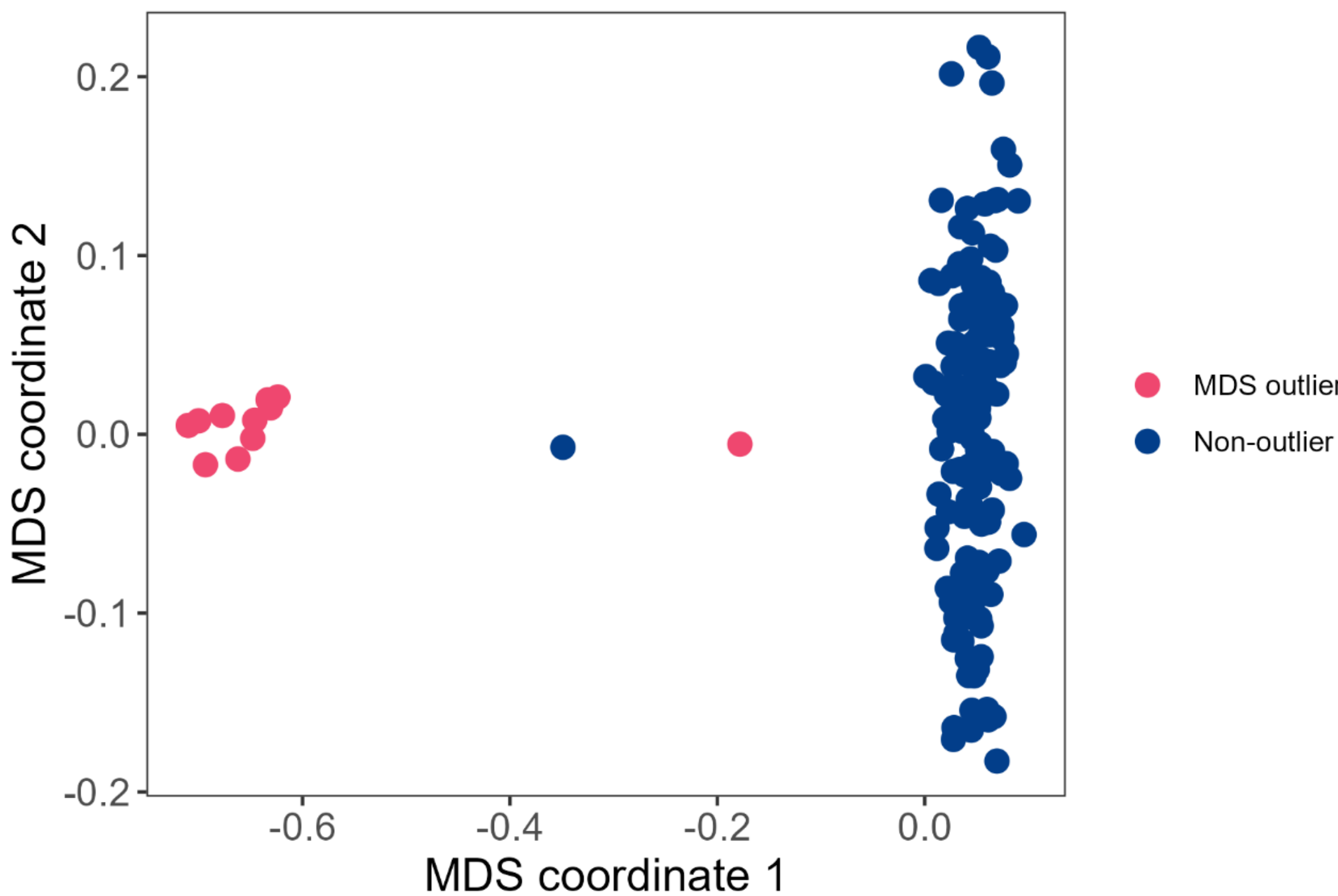

Figure S9. MDS plot from a local PCA using 50 SNP windows (lostruct) of chromosome XXI. MDS outlier windows are shown in pink and neutral windows are shown in blue. The MDS outlier windows correspond to the outlier regions on chromosome XXI that differentiate geothermal and ambient ecotypes in parallel.

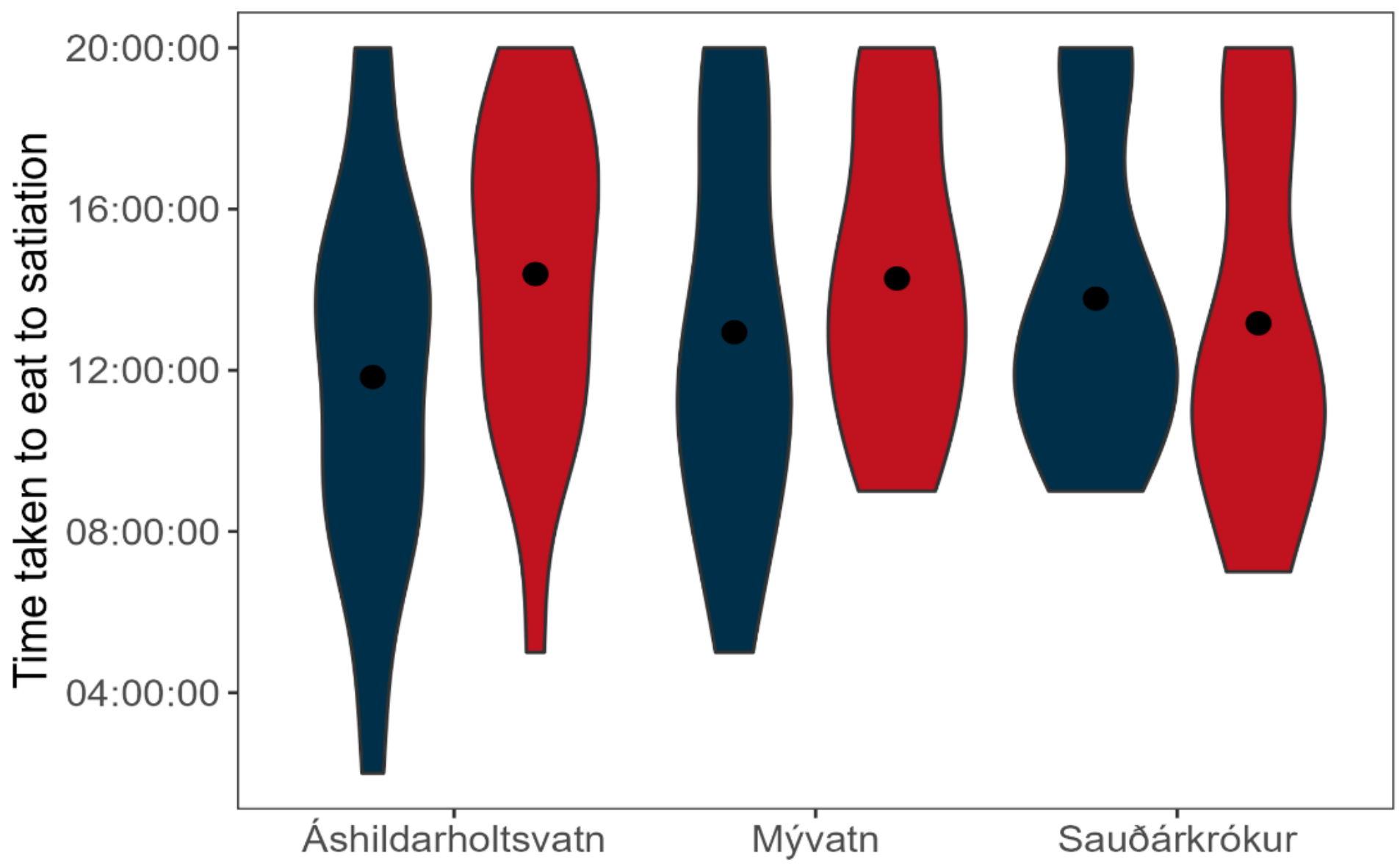

Figure S10. Evaluation of the time taken to eat pellets to satiation. Geothermal and ambient ecotypes are shown in red and blue, respectively. Geothermal and ambient ecotypes did not differ in the time taken to eat to satiation. There were no differences in time spent eating between ecotypes (df = 2, F-value = 1.29, p-value = 0.28).
