# Supplementary material for: Parallel adaptation to geothermally-warmed habitats due to common structural variation and functional developmental pathways
Source: Nat Commun. 2026 Jul 22;17:10103. doi: 10.1038/s41467-026-75785-0 (PMC13598157; doi:10.1038/s41467-026-75785-0)
Supplement: Supplementary file 2 [file 41467_2026_75785_MOESM2_ESM.pdf]

nature portfolio

Peer Review File

Parallel adaptation to geothermally-warmed habitats due to common structural variation and functional developmental pathways

Corresponding Author: Dr Matthew Brachmann

This file contains all reviewer reports in order by version, followed by all author rebuttals in order by version.

Version 0:

Reviewer comments:

Reviewer #1

(Remarks to the Author)
In this study, Dr. Brachmann and colleagues tried to understand genetic changes underlying rapid increases in temperature during climate change by using by comparisons of threespine sticklebacks between geothermal and ambient habitats. While the orgnisim is unique in terms of its recurrent adaptation to geothermal habitat, the novelties of concepts and findings in this study are limited. First, the parallel genetic and polygenic adapation to environmental or climative changes have been found in many various species. Second, the study has not shown functional (or experimental) evidence of how MAPK signalling pathway is key to the recurrent adaptation to geothermal habitat in threespine sticklebacks, but genetic changes in different genetic changes in different genes. Third, althrough threespine sticklebacks show parallel phenotypic differences between geothermal and ambient, which is consistent with genomic investigation, there is no straightforward link between the two study lines. Finally, the current evidence for chromosome XXI inversion driving ecological differentiation between geothermal and ambient habitats is insufficient. The presented analyses fail to conclusively demonstrate the proposed mechanism of structural variation facilitating thermal adaptation.

Additionally, there are some specific suggestions regarding the details of the manuscript.

The resolution of Figure 1A is insufficient, resulting in unclear differentiation among the clades in the phylogenetic tree. There is no clear distinction between ancient and modern populations, nor between marine and freshwater groups. While the manuscript suggests genetic similarity between two marine populations (sites 15-16), the current phylogenetic topology contradicts this assertion. Notably, sites 14-15 cluster together, while site 16 remains distinctly separated, thereby undermining the claimed genetic homogeneity. The differentiation of the oldest location, Grettislaug (site 6), from all other freshwater populations is not effectively represented in Figure 1A. The clearer depiction of this divergence is needed to support the manuscript’s claims regarding its genetic uniqueness. The current representation in Figure 1B is overly simplified. I recommend incorporating additional regional information, geographic coordinates (latitude and longitude) and key regional landmarks to enhance the contextual understanding of the sampling sites. A scale bar and clearer geographic reference points are needed in the sampling map. In the Treemix analysis results presented in Figures 1E and 1F, it is essential to include migration weight values. This addition will provide a clearer indication of the intensity of gene flow between populations, which is critical for interpreting the dynamics of genetic exchange.
Line 171-172：Both Figure 2 and Table 1 fail to clearly illustrate the impact of population age differences on thermal habitat differentiation. To enhance clarity, it is essential to implement statistical tests to quantify the correlation between population age and adaptive genetic variation. Additionally, I recommend developing a more informative visualization that explicitly connects population age, thermal habitat characteristics, and genetic differentiation would improve clarity and facilitate interpretation of the findings. The differences in the number of outlier loci among various populations are not well-defined, and the overlap between outlier loci is not adequately represented. It would be beneficial to apply statistical tests (e.g., Fisher’s exact tests or Jaccard indices) to rigorously compare outlier loci overlap between age-stratified populations. Including a Venn diagram or similar visualization could provide clearer insights into shared and unique outlier loci across populations. Furthermore, the statement regarding low parallelism in outlier loci is not sufficiently supported by the data presented in Table 1. To enhance the rigor of this analysis, it is recommended to incorporate additional statistical tests to quantitatively assess the significance of observed patterns in outlier loci.
Line 249: Supplementary Figure 5 should be renumbered as Supplementary Figure 4. Additionally, both Figure 4C and

Supplementary Figure 4 fail to pronounced allele frequency divergence on chromosome XXI. There is also an inconsistency in the labeling of chromosome names between Figure 4B and Figure 4C, which hinders accurate identification of chromosome XXI. Moreover, the most pronounced outliers in both figures do not correspond to chromosome XXI. Furthermore, the supplementary material lacks essential figure legends, making it difficult to discern the analytical differences between Figure 4C and Supplementary Figure 4. The methodological basis for comparing these figures remains unclear.
Line 250-256: The detection of an FST outlier within the 1.6 Mb region, along with elevated levels of XP-EHH and MDA outliers, suggests the potential presence of inversions or other structural variations. However, it is critical to supplement this analysis with principal component analysis (PCA) of this specific region. Currently, the presented results do not sufficiently demonstrate that this segment can effectively differentiate thermal ecotypes. Relying solely on local PCA to infer that structural variation has facilitated adaptation to temperature is insufficient. This approach does not clarify which populations exhibit structural variations or how differentiation occurs within this region. It should provide detailed analyses showing how structural variation is distributed across populations and the specific mechanisms by which this region contributes to thermal adaptation.
Line 253 and 255: There is an error in the labeling of the figures. Specificity, Figure 5B and Figure 5C appear to be switched. Please verify the figure assignments to ensure they are correctly matched to their respective descriptions in the manuscript.
Line 263: The description regarding the evolutionary origins of sticklebacks in the manuscript is inaccurate. Sticklebacks are derived from marine environments, not freshwater habitats (McKinnon and Rundle, 2002; Bell and Foster, 1994). The manuscript incorrectly suggests that the inversion on chromosome XXI has been associated with freshwater adaptation based on Dean (2024). However, as noted in this paper, this inversion does not effectively distinguish between marine and freshwater genomic groups since it is present in both populations. Therefore, according to the citation, it does not support the claim that the inversion is strongly associated with freshwater adaptation.
To substantiate the hypothesis that the inversion on chromosome XXI is related to thermal adaptation, the authors must provide further investigation into whether this inversion is exclusively or predominantly found in geothermal habitats, while absent or rare in ambient habitats. This distinction will be crucial in supporting claims regarding its role in thermal adaptation.

(Remarks on code availability)

Reviewer #2

(Remarks to the Author)
Review for “Parallel adaptation to geothermally-warmed habitats due to common structural variation and functional developmental pathways” by Brachmann et al.

Summary of paper:
Brachmann et al. examined the evolutionary responses of natural populations to increased temperatures using an interesting model of ambient and geothermal stickleback populations. The authors present evidence suggesting strong differentiation between ecotypes within each population, but low parallelism between independent ecotype pairs. They identify a potential structural mechanism for adaptation to increased temperatures: an inversion on chromosome XXI that is associated with ambient and geothermal ecotypes in these populations. The authors argue that structural variation and allelic divergence resulting in similar functional molecular pathways are both important for adaptation and should be considered in future conservation efforts.
Overall, the paper is well-written and presents a unique, direct investigation of adaptation to increasing temperatures, a topic that is often challenging to study. I have some general comments and questions regarding the interpretation of the results. This mostly concerns the GO enrichment analysis for the genomic differentiation and inversion, which I think sometimes overinterprets certain findings and is too speculative.

Comments:
- L29: The MAPK signaling pathway doesn’t appear in ecotype pair GTS-GAR.
- L81-84: There are some examples for the role of inversions in thermal adaptation in insects (Zivanovic, G., Arenas, C., & Mestres, F. (2014). Inbreeding and thermal adaptation in Drosophila subobscura. Genome, 57(9), 481-488. & Ma, L. J., Cao, L. J., Chen, J. C., Tang, M. Q., Song, W., Yang, F. Y., ... & Wei, S. J. (2024). Rapid and repeated climate adaptation involving chromosome inversions following invasion of an insect. Molecular biology and evolution, 41(3), msae044.)
- L94-96: What other changes in biotic and abiotic factors are found in geothermally warmed habitats? I imagine temperature is the most prominent factor, but are there others that affect sticklebacks? It would be interesting to discuss this here.
- L139-142: Why did you select the populations SKR, GAR, and ASHN, which clustered together in Figure 1A?
- Figure 1: The quality of the figure is too low, and some of the text appears blurry.
- Figure 1D: For the PCA, the populations are separated into ecotypes. It would be helpful to include this in the Admixture plot as well to identify any differences.
- Figure 1E&F: What is population CSWY? Was GAR not included in this analysis?
- L185-190: I think Figure 3 and its interpretation are difficult to follow. While I understand the commonalities you suggest for the enriched terms, I think this is very subjective and up to interpretation. The terms sound like they belong to broadly similar categories, such as nerve and muscle cells, but I still think there is a lot of variation across population pairs. For example, there isn’t a GO term that is shared by multiple populations. I recommend rewording this paragraph carefully so that the reader can more easily understand the similarities.

- L196-198: Please elaborate here, I don't understand the direct connection of muscles and nerves to sociability.
- L224-227: While this is true for the Icelandic threespine stickleback, I think you can't extend this to all species. The geothermal warming is much higher than predicted climate change.
- L228: It was unclear to me that the gene network analysis is part of Metascape. Please elaborate on this in the methods and explain what you mean by "gene network".
- L231: Do you have an explanation for why this is not found in GTS-GAR?
- Figure 4: I think you only need to show Figure 4B here, and Figure 4A can be included in the supplementary material.
- Figure 4C: For clarity, I think this should use the same stickleback chromosome names as the other plots, because chromosome XXI is now called 23.
- L250: Could you define the genomic coordinates of the inversion here? It would also be helpful to mark the inversion in Figure 5.
- L253,255: These figures should be switched.
- L276: Please specify which GO term relates to which term you use here. I'm not sure which one is morphology or epigenetic regulation. However, I think morphology is a very broad term and hard to identify based on a single GO term.
- L282-297: I think the interpretation of the specific function of metabolism and neddylation is very speculative. I don't know much about Mexican cave fish, but isn't the cave environment nutrient-limited year-round? So, I don't quite understand the connection between seasonal metabolic demands faced within geothermal habitats and the Mexican cave fish.
- L289-291: What are the temperatures in geothermal habitats in the winter? Wouldn't the increased temperature also increase food availability? Maybe you should support this with a citation.
- L303-307: I couldn't quite follow this reasoning. Is the PPARA gene also located in the inversion?
- L817: Which library kit was used?
- L885: What is the difference between parallel and multi-parallel?
- L900, 903: A similar sentence appears twice.
- L914-915: Since this is an important basis for differences between geothermal and ambient ecotypes, I think this should be more detailed. For example, what species was used, and what were the parameters?
- Figure 5D: It is not clear to me what the differences between the top and bottom panels are. Please explain what you mean by "biological function".
- Figure 6: There must have been logistical reasons for using a different combination of population pairs for the appetite trials, food restriction experiments, and body fat assessment, but you should discuss this choice and its potential effect on the results.
- Supplementary material: In the version I received, the legends are missing, and the table and figures are not numbered. Only two of the figures are referred to in the main text.

(Remarks on code availability)
The code is well-documented. It would be helpful to have a README file describing the function of each script. It seems that the script "Demographic_inference.R" is not relevant for this version of the manuscript.

Reviewer #3

(Remarks to the Author)
I co-reviewed this manuscript with one of the reviewers who provided the listed reports. This is part of the Nature Communications initiative to facilitate training in peer review and to provide appropriate recognition for Early Career Researchers who co-review manuscripts.

(Remarks on code availability)

Reviewer #4

(Remarks to the Author)
I co-reviewed this manuscript with one of the reviewers who provided the listed reports. This is part of the Nature Communications initiative to facilitate training in peer review and to provide appropriate recognition for Early Career Researchers who co-review manuscripts.

(Remarks on code availability)

Version 1:

Reviewer comments:

Reviewer #1

(Remarks to the Author)
General Comments
Despite the authors' efforts to address previous reviewer comments, specifically in the following five areas:
(1) Addition of common garden experiments and RNA-seq data
(2) PCA analysis of chromosome XXI inversion region

(3) Removal of speculative GO term analysis
(4) Removal of problematic TreeMix analysis
(5) Identification of MAPK pathway in all population pairs
The revisions do not sufficiently resolve the fundamental issues identified in the initial review. Furthermore, several important questions remain inadequately addressed.

1. Limited sample size
A sample size of just four population pairs may not adequately capture the breadth of genetic and phenotypic variation present in both geothermal and surface environments. Different populations can exhibit unique adaptations and evolutionary responses based on their specific ecological contexts, and the restriction to only these pairs limits the generalizability of the results. Each population's ecological history and genetic background can significantly influence adaptive traits. A broader sampling strategy that encompasses more diverse populations from both geothermal and surface habitats would provide a more comprehensive understanding of the adaptive mechanisms at play. With such a small sample size, the statistical analyses performed may lack sufficient power to detect meaningful patterns and differences between groups. Smaller sample sizes can lead to increased variability in results, making it difficult to draw robust conclusions regarding adaptation. In summary, while the study provides intriguing insights into the adaptations of sticklebacks in geothermal environments, the limited representativeness of the chosen population pairs notably constrains the applicability of the findings across broader ecological contexts.

2. Insufficient functional evidence
Although authors have included common garden experiments and RNA-seq data to support the role of the MAPK signaling pathway, the functional evidence remains weak. Authors have improved the logical connection between genotype and phenotype, but this remains an inferential link rather than a direct demonstration. The connection between the MAPK pathway and the observed phenotypic adaptations is not adequately demonstrated. While the presence of MAPK genes was established, the direct functional implications of these genes in the context of thermal adaptation are still unclear. The lack of direct experimental validation of the proposed mechanisms undermines the strength of conclusions. The authors should acknowledge this limitation explicitly in the Discussion section, framing the mechanistic pathway as a well-supported hypothesis that warrants future experimental validation.
3. Insufficient evidence for chromosome XXI inversion driving ecological differentiation
The manuscript presents a PCA analysis of the chromosome XXI inversion but does not convincingly demonstrate that this structural variation is responsible for thermal adaptation. The authors acknowledge that the full functional role of the inversion is not understood, and the evidence provided does not adequately support the claim that it facilitates adaptation to geothermal environments. The analyses presented are insufficient to establish a clear mechanism linking the inversion to ecological differentiation.

4. Limited representation and contribution to the field
While this study presents interesting findings regarding the adaptation of threespine sticklebacks to geothermal habitats, the novelty of the concepts and findings is limited. The authors make a valid point that their system provides insight into populations that have successfully adapted to warming, which is valuable for understanding adaptive potential. However, the magnitude difference between geothermal warming and predicted climate change (order of magnitude difference) remains a legitimate caveat that deserves more explicit acknowledgment. The authors should add a clear statement in the Discussion acknowledging this limitation. For example: "While the temperature differential in our system (~15-20°C) exceeds near-term climate change projections, our findings identify developmental pathways that may represent conserved targets of thermal adaptation. Future studies in systems with more moderate temperature differentials will be important for assessing the generality of these findings."
5. Speculative interpretation of metabolism and neddylation
The parallel between Icelandic geothermal stickleback and Mexican cavefish is conceptually interesting but the systems differ in important ways. The cavefish system involves extreme, constant resource limitation, whereas the geothermal stickleback system involves seasonal resource fluctuation with elevated metabolic demands. Clearly label speculative interpretations as hypotheses. Consider adding language such as: "While speculative, the convergent metabolic signatures suggest that thermal adaptation may involve similar physiological solutions across diverse systems, though the specific ecological pressures differ."

6. Different population combinations across experiments
While logistical constraints are understandable and the statistical consistency across pairs is reassuring, this experimental heterogeneity should be acknowledged as a limitation. The authors have attempted to address the reviewers' concerns regarding statistical analyses, including the use of Fisher’s exact tests and Sorensen-Dice similarity indices. However, the results remain poorly defined, and the statistical methods employed do not adequately support the claims made regarding the significance of outlier loci and their relationship to population age and adaptation. The lack of clear and rigorous statistical testing undermines the reliability of the findings. Add a brief statement in the Methods or Results section acknowledging this limitation. For example: "Due to logistical constraints, different population combinations were used across phenotypic experiments. However, results were statistically consistent across all tested ecotype pairs, and three populations (MYV, ASHN, SKR) appeared in all experiments, providing internal replication."

Conclusion
In summary, while the authors have made commendable efforts to address previous reviewer comments, the manuscript ultimately fails to provide sufficient evidence to support its central claims. The lack of functional evidence, insufficient statistical rigor, and speculative interpretations significantly weaken the study's contributions to the field of evolutionary biology and climate adaptation. Therefore, I recommend rejecting the manuscript in its current form.

(Remarks on code availability)

Reviewer #2

(Remarks to the Author)
Review for “Parallel adaptation to geothermally-warmed habitats due to common structural variation and functional developmental pathways” by Brachmann et al.

I feel that the authors have addressed the concerns of both the other reviewer and myself and have improved the manuscript. They have clarified important points and strengthened their interpretation of the GO enrichment analysis. In addition, they present further evidence of the role of the inversion on chromosome XXI and common garden experiments that support the role of the mapk signaling pathway in geothermal adaptation. I have a few minor comments regarding the new sections.

Comments:

- L178-179, Figure 2E: I think the figures work well to compare the levels of parallelism, but I’m unsure how you calculated the predicted degree of genomic parallelism. Please elaborate on this in the methods.

- L264-267: I’m having trouble understanding how these Fst values are elevated and point to the braf gene being under selection. These values seem to be close to the average Fst (L164) and don’t seem to be classified as outliers in 2A-D.

- L1169-1174: Why did you decide to use enrichR in addition to Metascape for the gene ontology analysis? I assume that the new results in figure 3B and figure 4F use this method, but this is not clear to me from the manuscript. Please clarify which method you used in each analysis. In addition, what is the meaning of the colors in these figures? In Figure 4F, I find the GO terms harder to understand than before, and I can’t quite follow your interpretation that the genes in the inversion are related to metabolism, hypoxia, and cellular repair (L315).

- Figure 4B: I think it’s helpful to have this figure differentiating the three potential states of the inversion. Please explain what the different shapes mean and the ellipses seem to exclude some samples. However, since the coordinates of the geothermal-ambient inversion overlap with the inversion found in all freshwater populations, I was wondering how you can be sure that the homozygous cluster most common for the geothermal populations is the inverted karyotype and not the ancestral karyotype, while the ambient homozygous cluster is the inversion (L297-304)?

- L1098-1011: After removing the Treemix figure, you should also remove the corresponding methods here.

- Many of the supplementary figures are not mentioned in the main text and I would carefully review them to ensure they are all still relevant for the revised manuscript.

- L1239-1251: The methods seem to be very similar for both common garden experiments, so I would remove this and mention that it was performed as described above.

- Figure 6 should be labelled as figure 5.

(Remarks on code availability)

Reviewer #3

(Remarks to the Author)
I co-reviewed this manuscript with one of the reviewers who provided the listed reports. This is part of the Nature Communications initiative to facilitate training in peer review and to provide appropriate recognition for Early Career Researchers who co-review manuscripts.

(Remarks on code availability)

Reviewer #4

(Remarks to the Author)
I co-reviewed this manuscript with one of the reviewers who provided the listed reports. This is part of the Nature Communications initiative to facilitate training in peer review and to provide appropriate recognition for Early Career Researchers who co-review manuscripts.

(Remarks on code availability)

Version 2:

Reviewer comments:

Reviewer #1

(Remarks to the Author)
Thanks the authors for your efforts to exclude my concerns. I think that I am now convinced of their explaination to limited sample size and insufficient functional evidence. Particularly, thanks for their statements of limitations in related discussion.

(Remarks on code availability)

Reviewer #2

(Remarks to the Author)
Review for “Parallel adaptation to geothermally-warmed habitats due to common structural variation and functional developmental pathways” by Brachmann et al.
I have assessed this manuscript in both rounds and I think the authors have made significant improvements. I am satisfied with how they addressed my previous comments, and I appreciate their clear explanations and added clarifications. While the functional role of the inversion and its ecological differentiation, as well as the link to MAPK signaling, are not yet fully understood, I feel the authors describe these aspects with appropriate caution. I particularly like the “A way forward for evolution” paragraph, which nicely ties the manuscript together, and overall, I think this work provides a valuable system to study thermal adaptation.

(Remarks on code availability)
I signed off on the pevious draft of the manuscript and have no additional comments.

Reviewer #3

(Remarks to the Author)
I co-reviewed this manuscript with one of the reviewers who provided the listed reports. This is part of the Nature Communications initiative to facilitate training in peer review and to provide appropriate recognition for Early Career Researchers who co-review manuscripts.

(Remarks on code availability)

Reviewer #4

(Remarks to the Author)
I co-reviewed this manuscript with one of the reviewers who provided the listed reports. This is part of the Nature Communications initiative to facilitate training in peer review and to provide appropriate recognition for Early Career Researchers who co-review manuscripts.

(Remarks on code availability)

Open Access This Peer Review File is licensed under a Creative Commons Attribution 4.0 International License, which permits use, sharing, adaptation, distribution and reproduction in any medium or format, as long as you give appropriate credit to the original author(s) and the source, provide a link to the Creative Commons license, and indicate if changes were made.
In cases where reviewers are anonymous, credit should be given to 'Anonymous Referee' and the source.
The images or other third party material in this Peer Review File are included in the article's Creative Commons license, unless indicated otherwise in a credit line to the material. If material is not included in the article's Creative Commons license and your intended use is not permitted by statutory regulation or exceeds the permitted use, you will need to obtain permission directly from the copyright holder.
To view a copy of this license, visit https://creativecommons.org/licenses/by/4.0/

RESPONSE TO REVIEWERS' COMMENTS

We are pleased to provide a revised version of our manuscript 'Parallel adaptation to geothermally-warmed habitats due to common structural variation and functional developmental pathways' for your consideration after major revisions. We would like to thank both reviewers for their comments and suggestions on the previous version of our manuscript. We feel that the suggestions raised by both reviewers have elevated the quality and impact of the revised manuscript. Throughout the major revisions of this manuscript, we have addressed all comments and suggestions made by both reviewers.

To address the concerns of the reviewers, we have now added in two common garden experiments (performed by Dr. Bethany Smith) to better understand the functional role for the *mapk* signalling pathway in thermal adaptation. These common garden experiments provided gene expression data for the *mapk* pathway and allowed for a better understanding of genotype-by-environment interactions of key genes in the pathway as well as their contribution to reproductive isolation between the ecotypes.

Additionally, we have provided further evidence for the ecological role of the inversion on chromosome XXI in thermal adaptation. We believe the revisions provide sufficient evidence to support a key role for the inversion in parallel adaptation to contrasting thermal environments. Furthermore, we have now specifically highlighted the novelty of this study within the revised manuscript. We believe our research highlights a combined role for a polygenic genomic basis and structural variation in facilitating adaptation to novel environmental conditions.

Overall, the major revisions to the manuscript suggested by both reviewers have substantially improved the manuscript.

We thank you for your time and look forward to your reply.

Sincerely,

Matthew K. Brachmann

Kevin J. Parsons

Reviewer #1 (Remarks to the Author):

In this study, Dr. Brachmann and colleagues tried to understand genetic changes underlying rapid increases in temperature during climate change by using by comparisons of threespine sticklebacks between geothermal and ambient habitats. While the orgnisim is unique in terms of its recurrent adaptation to geothermal habitat, the novelties of concepts and findings in this study are limited.

First, the parallel genetic and polygenic adapation to environmental or climative changes have been found in many various species.

Response: While we agree to a point with reviewer 1 our study presents novelty in three key ways:

1. While the parallel divergence of phenotypic and ecological variation has been identified previously, evidence for a correspondingly parallel genomic basis is not a general phenomenon. Contrary to expectation it is often the case that the genomic basis for adaptation is highly non-parallel across divergent populations (**lines 178-187**). Therefore, identifying a parallel basis for adaptation at any level of genomic organization represents significant novelty.
2. We identified non-parallel polygenic adaptation to geothermal environments, in addition to a parallel genomic basis that occurs through structural variation. While we agree that polygenic adaptation is not a novel concept, critically we have identified a novel outcome in which both non-parallel polygenic divergence and parallel structural variation **occur simultaneously.** Generally, polygenic adaptation and structural genomic variation are studied in isolation and thus there has not been an opportunity to link these phenomena. Our novel findings provides important novel insight into our understanding of the genomic basis of adaptation. We have amended the text from **lines 367-401** to better clarify these points.

Furthermore, as mentioned in our manuscript (**lines 57-73; 87-95**), the majority of research on the genomic basis of climate adaptation has occurred along large latitudinal gradients or through employing genomic offset modelling. These approaches can be open to criticism because latitudinal gradients vary in a suite of environmental factors that are confounded with temperature, while genomic offset models make very strong assumptions of adaptation (Lotterhos, 2024; https://doi.org/10.1093/evlett/qrae004). We believe our study is leveraging a system which provides unprecedented clarity beyond previous research, given the simplicity of the habitats. Indeed, our environmental data from these systems (https://doi.org/10.1093/evolut/qpac018) highlights similarity across populations in environmental variables aside from temperature. Therefore, we believe we have now provided some of the most reliable insights into how thermal variation drives adaptation.

Second, the study has not shown functional (or experimental) evidence of how MAPK signalling pathway is key to the recurrent adaptation to geothermal habitat in threespine sticklebacks, bsut genetic changes in different genetic changes in different genes.

Response: To address this important point we now include data from common garden experiments combined with RNA-seq data that directly addresses this (**line 237-277; line 1175**).These new findings demonstrate heritable divergence and plasticity in gene expression in response to temperature across population pairs. Specifically, the RNA-seq analysis demonstrates ecotype effects on the *mapk* signalling pathway from two separate common garden experiments (**Figure 3**). These new analyses demonstrates a functional response to temperature specific to the *mapk* pathway that is present across all population pairs. Thus, as our previous draft showed a presence of *mapk* in all population pairs, this new gene expression data strongly reinforces our previous findings indicated from allelic variation.

Specifically, significant ecotype-temperature interactions were found across population pairs. We also found that hybridization substantially alters the function of the *mapk* pathway, likely

in a maladaptive way which suggests a mechanism for the maintenance of reduced gene flow. Details of these new findings can be found from **line 252-277**.

Third, althrough threespine sticklebacks show parallel phenotypic differences between geothermal and ambient, which is consistent with genomic investigation, there is no straightforward link between the two study lines.

Response: As reviewer 1 points out, the parallel phenotypic differences between geothermal and ambient ecotypes is consistent with the genomic results that we have presented. An inversion on chromosome XXI is clearly associated with temperature adaptation and some genes within the inversion play a role in metabolic adaptation. However, we agree that the full functional role of this inversion is still not fully understood. As we explained on **lines 339-347**, the *ncoa2* gene within the inversion interacts with the well characterized *ppar* pathway which in turn connects to the *mapk* signalling pathway. Thus, the genomic results provide plausible predictions for how phenotypes should respond between geothermal and ambient ecotypes. We then test these predictions using a mix of lab reared and wild ecotypes.

Finally, the current evidence for chromosome XXI inversion driving ecological differentiation between geothermal and ambient habitats is insufficient. The presented analyses fail to conclusively demonstrate the proposed mechanism of structural variation facilitating thermal adaptation.

Response: To address this concern we have conducted a PCA of the putatively inverted region of chromosome XXI in our revision (**Figure 4B**). From a population genomic perspective, a PCA of an inversion typically shows three clusters that correspond to the different inversion karyotypes. Our PCA clearly shows that geothermal ecotypes are dispersed between all three karyotypes with the majority being in the inverted homokaryotype, while the ambient ecotypes are rarely in the inverted homokaryotype (**Line 283-287; Figure 4B**). This region also differentiates the geothermal and ambient ecotypes based on cross population selection coefficients (**Figure 4E**). These methodological approaches are standard in the field including journals within the Nature family (Todesco et al., 2020, doi.org/10.1038/s41586-020-2467-6; Harringmeyer and Hoekstra, 2022, doi.org/10.1038/s41559-022-01890-0).

Additionally, there are some specific suggestions regarding the details of the manuscript.

The resolution of Figure 1A is insufficient, resulting in unclear differentiation among the clades in the phylogenetic tree. There is no clear distinction between ancient and modern populations, nor between marine and freshwater groups. While the manuscript suggests genetic similarity between two marine populations (sites 15-16), the current phylogenetic topology contradicts this assertion. Notably, sites 14-15 cluster together, while site 16 remains distinctly separated, thereby undermining the claimed genetic homogeneity. The differentiation of the oldest location,

Grettislaug (site 6), from all other freshwater populations is not effectively represented in Figure 1A. The clearer depiction of this divergence is needed to support the manuscript's claims regarding its genetic uniqueness. The current representation in Figure 1B is overly simplified. I recommend incorporating additional regional information, geographic coordinates (latitude and longitude) and key regional landmarks to enhance the contextual understanding of the sampling sites. A scale bar and clearer geographic reference points are needed in the sampling map. In the Treemix analysis results presented in Figures 1E and 1F, it is essential to include migration weight values. This addition will provide a clearer indication of the intensity of gene flow between populations, which is critical for interpreting the dynamics of genetic exchange.

Response: We have remade the phylogenetic tree to be clearer and more informative (see **Figure 1A**). There is now a clear distinction between geothermal, ambient, and marine ecotypes of Icelandic stickleback. We however maintain that there is a clear grouping between both marine populations, but the poor resolution of the previous version meant that the figure was unable to show this effectively. The Grettislaug (GTS) population and its relationship to the other stickleback populations is now also shown more clearly in **Figure 1A**. In addition, to further clarify this we have now included ancestry proportions to identify admixture among the 25 stickleback populations in **Figure 1B**.

As we have previously published a map of our study population in Evolution (Pilakouta et al., 2023, https://doi.org/10.1093/evolut/qpac018), we have opted to remove the map of Iceland. As in the previous version of the manuscript, all information on the sampling locations are provided in supplementary table 1 (**Table S1**). This change also allows us to better highlight the large phylogeny in **Figure 1A**.

We agree with reviewer 1 about the TreeMix figures. However, upon review of TreeMix (https://doi.org/10.1371/journal.pgen.1002967), our data appears to violate two major assumption of the program as we have a high degree of ongoing gene flow and three of our populations are not tree-like. Therefore, as the conclusions may be unreliable we have opted to remove the TreeMix figures. We have instead opted to add a phylogenetic tree (**see figure 1E**) based on the whole genome sequencing data, which we believe more clearly shows the relationships among stickleback populations.

Line 171-172：Both Figure 2 and Table 1 fail to clearly illustrate the impact of population age differences on thermal habitat differentiation. To enhance clarity, it is essential to implement statistical tests to quantify the correlation between population age and adaptive genetic variation. Additionally, I recommend developing a more informative visualization that explicitly connects population age, thermal habitat characteristics, and genetic differentiation would improve clarity and facilitate interpretation of the findings. The differences in the number of outlier loci among various populations are not well-defined, and the overlap between outlier loci is not adequately represented. It would be beneficial to apply statistical tests (e.g., Fisher's exact tests or Jaccard indices) to rigorously compare outlier loci overlap between age-stratified populations. Including a Venn diagram or similar visualization could provide clearer insights into shared and unique outlier loci across populations. Furthermore, the statement regarding low parallelism in outlier loci is not sufficiently supported by the data presented in Table 1. To enhance the rigor of this analysis, it is

recommended to incorporate additional statistical tests to quantitatively assess the significance of observed patterns in outlier loci.

Response: We have now **updated figure 2** to better illustrate the lack of parallelism of outlier loci and genes differentiated ecotype pairs (**line 177-184**). In the new version of figure 2, we have included Venn diagram of predicted parallelism given the geographic population structure as well as time since divergence of each population (**Figure 2E**). This provides a clear contrast of what is expected and what the real data show biologically in **figure 2 (F-G)**. Our findings suggest that population age does not impact out results as the youngest ecotype pair (SKR, ~70 years old) and the oldest ecotype pair (GTS-GAR, ~10,000 years old) share the greatest number of Fst outliers as well as genes containing Fst outliers. If population age did have an effect, based on the population structure shown in **figure 1 and figure 2E**, we would expect to see much greater sharing of differentiated loci and genes between SKR and ASHN as they are indistinguishable phylogenetically and physically adjacent. Instead we see sharing between clearly defined ecotype pairs and populations, counter to the effects of population age and demography.

We politely disagree with reviewer 1, as population age did not influence our results. **Figure 2 F&G** (Table S2) shows that the number of shared Fst outlier loci and genes between each ecotype pair are extremely low especially given the number of total outlier loci and genes found differentiating each ecotype pair. To address this formally, we calculated a Sorensen-dice similarity index (see below), as it weighs shared loci and genes more heavily making it more conservative, with the formula: (2 * shared_loci) / (total_pop1 + total_pop2). The results mirror the results we have already presented in **Figure 2 F&G and Table S2** (see below this response). The youngest and oldest ecotype pairs have a greater index value than any of the other ecotype pairs. We have not added this index into the manuscript as using a formula to compare similarity when the number of shared outliers is at such an extreme low obscures the main point and would add unnecessary complexity to the manuscript.

	**ASHN**	**MYV**	**SKR**	**GTS-GAR**
**ASHN**	-	0	0.0024	0.0023
**MYV**	0	-	0.0097	0.0047
**SKR**	0.0035	0.0036	-	0.0067
**GTS-GAR**	0.0032	0.0065	0.0095	-

In line with the reviewers comments, we have now performed Fisher's exact tests (**line 1110 and 1113**) and the results show that all ecotype pairs do not share outlier loci or genes (all ecotype pairs

have unique outlier loci); see **lines 177 and 184**. Between all ecotype pairs the number of shared outliers is extremely low compared to the number of unique outlier loci in each of the ecotype pairs.

Line 249: Supplementary Figure 5 should be renumbered as Supplementary Figure 4. Additionally, both Figure 4C and Supplementary Figure 4 fail to pronounced allele frequency divergence on chromosome XXI. There is also an inconsistency in the labeling of chromosome names between Figure 4B and Figure 4C, which hinders accurate identification of chromosome XXI. Moreover, the most pronounced outliers in both figures do not correspond to chromosome XXI. Furthermore, the supplementary material lacks essential figure legends, making it difficult to discern the analytical differences between Figure 4C and Supplementary Figure 4. The methodological basis for comparing these figures remains unclear.

Response: The supplementary figures have been relabeled as requested. The chromosome names on all plots with multiple chromosomes have been re-labeled to be consistent. We agree that the most pronounced outliers across the genome do not correspond to chromosome XXI. Outlier loci that are not within the inversion region of chromosome XXI are also aiding in adaptation to geothermal and ambient habitats. While supplementary figure 5 (Fst in a 50 SNP window) does not show pronounced allele frequency divergence on chromosome XXI, supplementary figure 6 (per locus Fst) does show allele frequency divergence on chromosome XXI. There is a pronounced peak of Fst on chromosome XXI within the genomic region identified by the cross-population analysis (AFvaper); We show this region in more detail in **Figure 4D**.

Line 250-256: The detection of an FST outlier within the 1.6 Mb region, along with elevated levels of XP-EHH and MDA outliers, suggests the potential presence of inversions or other structural variations. However, it is critical to supplement this analysis with principal component analysis (PCA) of this specific region. Currently, the presented results do not sufficiently demonstrate that this segment can effectively differentiate thermal ecotypes. Relying solely on local PCA to infer that structural variation has facilitated adaptation to temperature is insufficient. This approach does not clarify which populations exhibit structural variations or how differentiation occurs within this region. It should provide detailed analyses showing how structural variation is distributed across populations and the specific mechanisms by which this region contributes to thermal adaptation.

Response: We have now incorporated a principal component analysis (PCA) on the 1.6 Mb inversion region that is differentiated the ecotypes in parallel (**Line 283 and 1154; Figure 4B**) as well as k-mean clustering (**line 1155**) to identify the three clusters that are characteristic of inversions. The PCA clearly shows that most geothermal ecotypes lie within a single cluster. While the local PCA approach is a valid approach to identifying putative inversions, the XP-EHH statistic identifies extended haplotypes across populations to identify regions under selection common to all geothermal and ambient ecotype pairs. As stated in the methods (**lines 1150-1151**), XP-EHH values that are more positive are associated with geothermal ecotypes while negative values are associated with ambient ecotypes. The inverted region is associated with the geothermal ecotype.

Line 253 and 255: There is an error in the labeling of the figures. Specificity, Figure 5B and Figure 5C

appear to be switched. Please verify the figure assignments to ensure they are correctly matched to their respective descriptions in the manuscript.

Response: We would like to thank reviewer 1 for pointing out this mistake. All figure assignments have been verified and this error has been corrected.

Line 263: The description regarding the evolutionary origins of sticklebacks in the manuscript is inaccurate. Sticklebacks are derived from marine environments, not freshwater habitats (McKinnon and Rundle, 2002; Bell and Foster, 1994). The manuscript incorrectly suggests that the inversion on chromosome XXI has been associated with freshwater adaptation based on Dean (2024). However, as noted in this paper, this inversion does not effectively distinguish between marine and freshwater genomic groups since it is present in both populations. Therefore, according to the citation, it does not support the claim that the inversion is strongly associated with freshwater adaptation.
To substantiate the hypothesis that the inversion on chromosome XXI is related to thermal adaptation, the authors must provide further investigation into whether this inversion is exclusively or predominantly found in geothermal habitats, while absent or rare in ambient habitats. This distinction will be crucial in supporting claims regarding its role in thermal adaptation.

Response: We would like to thank reviewer 1 for pointing out a couple of confusing sentences, we have now clarified this section of the manuscript. We had no intention of stating that freshwater sticklebacks were not derived from marine environments and apologise for any confusion. However, the geothermal stickleback in our study were most likely derived from freshwater populations after the invasion from the marine habitat (**Figure 1A**). The chromosomal inversion on chromosome XXI was shown to be associated with the marine-freshwater transition in Jones et al. (2012). This idea persists as show in recent research such as Dean et al. (2024) and Rodríguez-Fuentes et al. (2025), however these works show that this chromosome inversion is not related to salinity. Based on the PCA (**Figure 4B**), the inverted homokaryotype is rare in the ambient ecotype and dominated by the geothermal ecotypes. Thus, we have shown this inversion to be associated with thermal adaptation.

Reviewer #2 (Remarks to the Author):

Review for "Parallel adaptation to geothermally-warmed habitats due to common structural variation and functional developmental pathways" by Brachmann et al.

Summary of paper:
Brachmann et al. examined the evolutionary responses of natural populations to increased temperatures using an interesting model of ambient and geothermal stickleback populations. The authors present evidence suggesting strong differentiation between ecotypes within each population, but low parallelism between independent ecotype pairs. They identify a potential structural mechanism for adaptation to increased temperatures: an inversion on chromosome XXI that is associated with ambient and geothermal ecotypes in these populations. The authors argue that structural variation and allelic divergence resulting in similar functional molecular pathways are both important for adaptation and should be considered in future conservation efforts.

Overall, the paper is well-written and presents a unique, direct investigation of adaptation to increasing temperatures, a topic that is often challenging to study. I have some general comments and questions regarding the interpretation of the results. This mostly concerns the GO enrichment analysis for the genomic differentiation and inversion, which I think sometimes overinterprets certain findings and is too speculative.

Comments:

- L29: The MAPK signaling pathway doesn' t appear in ecotype pair GTS-GAR.

Response: We would like to thank the reviewer for this comment, as deeper investigations into the pathway, as prompted by reviewer 1, have generally made this manuscript stronger. We have now identified a role for the *mapk* pathway in the GTS-GAR ecotype pair. The divergent genes *myb* and *pppc3b* identified in the network analysis for the GTS-GAR ecotype pair are part of the *mapk* signalling pathway. The role for the mapk pathway in the GTS-GAR ecotype pair was identified following the suggestions made by reviewer 1 to provide a deeper functional role for the signalling pathway.

- L81-84: There are some examples for the role of inversions in thermal adaptation in insects (Zivanovic, G., Arenas, C., & Mestres, F. (2014). Inbreeding and thermal adaptation in Drosophila subobscura. Genome, 57(9), 481-488. & Ma, L. J., Cao, L. J., Chen, J. C., Tang, M. Q., Song, W., Yang, F. Y., ... & Wei, S. J. (2024). Rapid and repeated climate adaptation involving chromosome inversions following invasion of an insect. Molecular biology and evolution, 41(3), msae044.)

Response: We have added the citation provided by reviewer 2 into the manuscript (**line: 86**).

- L94-96: What other changes in biotic and abiotic factors are found in geothermally warmed habitats? I imagine temperature is the most prominent factor, but are there others that affect sticklebacks? It would be interesting to discuss this here.

Response: This is a good point. In terms of other abiotic factors, a previous paper indicates water chemistry results are fairly similar across populations (https://doi.org/10.1093/evolut/qpac018). We have not yet identified other abiotic factors differentiating the geothermal and ambient habitats. The geothermal and ambient habitats could however differ in biotic factors such as prey availability, predation, and microbial communities. We now discuss this on **lines 104-109** and micro-biome research is currently being done to explore this further. The MYV and ASHN populations do have other fish species (Arctic charr) present within them however, we do not know if they utilize geothermal habitats.

- L139-142: Why did you select the populations SKR, GAR, and ASHN, which clustered together in Figure 1A?

Response: While the phylogenetic relationships of these populations are highly similar, they differ substantially in their demographic history and population age. For whole genome sequencing data we wanted populations with geothermal and ambient ecotypes but also varied demographic

histories and ages. We aimed to identify the genomic regions differentiated the ecotypes without the confounding effects of demography and age. We now clarify this on **lines 1062-1064**.

- Figure 1: The quality of the figure is too low, and some of the text appears blurry.

Response: We have fixed the quality and overall representation of this figure, see new **Figure 1A**.

- Figure 1D: For the PCA, the populations are separated into ecotypes. It would be helpful to include this in the Admixture plot as well to identify any differences.

Response: The PCA in Figure 1D (**Figure 1C** in the new version) shows that the populations are separating by geography and not by ecotype. Separating the admixture plot by ecotype does not really add anything significant to the manuscript as the three ecotype pairs appear as a big mix of admixture. Please see new **Figure 1E** that highlights the relationships among these three populations.

- Figure 1E&F: What is population CSWY? Was GAR not included in this analysis?

Response: We thank the reviewer for pointing out an error in the figure. CSWY is a different name for the GAR population. We have however removed the treemix figures as we believe they do not add to the manuscript.

- L185-190: I think Figure 3 and its interpretation are difficult to follow. While I understand the commonalities you suggest for the enriched terms, I think this is very subjective and up to interpretation. The terms sound like they belong to broadly similar categories, such as nerve and muscle cells, but I still think there is a lot of variation across population pairs. For example, there isn’t a GO term that is shared by multiple populations. I recommend rewording this paragraph carefully so that the reader can more easily understand the similarities.

Response: We thank reviewer 2 for letting us know that the text needs to be clarified further. In the new version of the manuscript, we have removed the GO term section from the manuscript and streamlined the manuscript to focus on the *mapk* pathway (**line 237**). The *mapk* pathway was common to all geothermal and ambient ecotype pairs (stemming from the GO term analysis) while the actual GO terms were confusing and speculative. We have also included new data (**lines 237-277**) to show the function of the *mapk* pathway in promoting ecotype divergence based on two separate common garden experiments. This allows the manuscript to rely less on parallel GO terms and identify functional changes in the pathway of interest. This has helped to streamline the manuscript.

- L196-198: Please elaborate here, I don’t understand the direct connection of muscles and nerves to sociability.

Response: We thank the reviewer for identifying this, upon reflection we have removed this section from the text in the revision.

- L224-227: While this is true for the Icelandic threespine stickleback, I think you can' t extend this to all species. The geothermal warming is much higher than predicted climate change.

Response: We agree that the geothermal warming is higher than warming predicted by climate change. However, unlike current species that are yet to experience the full effects of climate change (and potentially adapt to it), Icelandic stickleback have found a way to adapt to the effects of warm water. Closely related populations diverging along a geothermal-ambient axis are doing so based on divergence in common functional pathways, not specific genes identified by population genomic approaches. As we stated on **lines 214-218**, we believe that targeting important developmental pathways, rather than specific loci, could be more broadly applicable to a variety of species facing changing climatic conditions.

- L228: It was unclear to me that the gene network analysis is part of Metascape. Please elaborate on this in the methods and explain what you mean by "gene network".

Response: Metascape performs a test for protein-protein interactions and identifies genes that form a network to form these protein interactions. This provides a list of genes that more than likely interact to form functional differences between the ecotypes. We have since supplemented this information with RNA-seq data from common garden experiments (see **lines 237 and 1175**) to highlight the role that the MAPK pathway is playing in ecotype divergence.

- L231: Do you have an explanation for why this is not found in GTS-GAR?

Response: This is a good question, and something that puzzled us. However, further analysis has now revealed genes involved in the *mapk* pathways (via KEGG) for this population pair, this includes *myb* and *ppp3cb*. The *mapk* pathway appears to play a role in geothermal-ambient divergence across all ecotype pairs. Based on comments from reviewer 1, this has now been followed up by two common garden experiments to confirm the role of the *mapk* pathway and its ecological relevance.

- Figure 4: I think you only need to show Figure 4B here, and Figure 4A can be included in the supplementary material.

Response: We agree and have moved both Figure 4A and 4B into the supplementary material. We now show pairwise Fst between all geothermal and ambient ecotypes for chromosome XXI in **Figure 4D** (new version).

- Figure 4C: For clarity, I think this should use the same stickleback chromosome names as the other plots, because chromosome XXI is now called 23.

Response: We agree with reviewer 2, the chromosome names have been updated to reflect the naming convention in the stickleback genome.

- L250: Could you define the genomic coordinates of the inversion here? It would also be helpful to mark the inversion in Figure 5.

Response: We have now defined the coordinates of the inversion here (**line 281**). We have also marked the inversion in **Figure 4**.

- L253,255: These figures should be switched.

Response: We have now fixed this mistake in the text (see **lines 282-285**).

- L276: Please specify which GO term relates to which term you use here. I' m not sure which one is morphology or epigenetic regulation. However, I think morphology is a very broad term and hard to identify based on a single GO term.

Response: We agree that morphology is hard to define by a single GO term. However, later in the paragraph (**line 318**) we aligned our genomic coordinates with a stickleback review paper on QTL (Peichel & Marques, 2017) and show that a number of morphological QTL line up with this inversion region on chromosome XXI.

- L282-297: I think the interpretation of the specific function of metabolism and neddylation is very speculative. I don' t know much about Mexican cave fish, but isn' t the cave environment nutrient-limited year-round? So, I don' t quite understand the connection between seasonal metabolic demands faced within geothermal habitats and the Mexican cave fish.

Response: We agree with reviewer 2 that the function of neddylation is speculative. However, the metabolic demands of geothermal environments in a highly seasonal place like Iceland do, we believe, have parallels with the Mexican cave fish. Geothermal habitats are stable across the summer and winter seasons and lakes are very nutrient and resource limited in the winter for a period exceeding 8 months. As geothermal habitats raise the overall metabolic demand on the fish, they also must get through a prolonged period of resource and nutrient limitation. It may not be as long as the Mexican Cavefish which have food available for approximately 2-3 weeks per year in the rainy season, but the general principals of low food and metabolic challenges still apply to the Icelandic system.

- L289-291: What are the temperatures in geothermal habitats in the winter? Wouldn' t the increased temperature also increase food availability? Maybe you should support this with a citation.

Response: We have included a citation showing that Icelandic lakes are resource poor in the winter (**lines 327-328**). The temperatures in the geothermal habitats are stable year round and can be greater than 20˚C in the winter, when resources are limited. We are currently conducting more work on the microbiome of these fish in connection with seasonal variation in resources. We also have a manuscript in preparation on the seasonal dynamics of resource availability in this system.

- L303-307: I couldn’t quite follow this reasoning. Is the PPARA gene also located in the inversion?

Response: The *ppara* gene is not located in the inverted region, the relationship between the genes and pathways have been clarified in the text (see **lines 337-359**). However, *ncoa2* and PPARA are a part of the same pathway that regulate adipogenesis. *Ncoa2* is a known coactivator of the *ppar* pathway and these two genes act to regulate adipogenesis and insulin sensitivity. The *ppar* pathway is also known to be related to the *mapk* pathway. The inversion more than likely alters the functioning, at least in part, of these pathways in the geothermal ecotype. This is a hypothesis that we indirectly test with the phenotypic experiments.

- L817: Which library kit was used?

Response: As stated, the library preparation was performed using a custom protocol rather than a kit.

- L885: What is the difference between parallel and multi-parallel?

Response: As per the AFvaper github page (https://github.com/JimWhiting91/afvaper) multi-parallelism is defined as ‘ Multi-parallel - Parallelism along multiple non-parallel axes, for e.g. 2 of 4 pops are parallel on eigenvector 1, and the other 2 pops are parallel on eigenvector 2.’

- L900, 903: A similar sentence appears twice.

Response: We have corrected the text on **lines 1112-1122**.

- L914-915: Since this is an important basis for differences between geothermal and ambient ecotypes, I think this should be more detailed. For example, what species was used, and what were the parameters?

Response: As Metascape does not have the threespine stickleback genome available. We converted gene ids to the homologous gene ids in the zebrafish genome. We have corrected the text on **lines 1171-1172** to make this clearer.

- Figure 5D: It is not clear to me what the differences between the top and bottom panels are. Please explain what you mean by “biological function”.

Response: GO terms are commonly split into three categories: Biological function, molecular function, and cellular function. The biological function category is the broadest and aids in identifying the function that each gene or set of genes plays.

- Figure 6: There must have been logistical reasons for using a different combination of population pairs for the appetite trials, food restriction experiments, and body fat assessment, but you should discuss this choice and its potential effect on the results.

Response: As the results are statistically consistent across all geothermal-ambient ecotype pairs, there is not an effect on the results or conclusions drawn from the phenotypic data (**line 357)**. These experiments were performed at different times (and years) and different populations were available for the experiments. The MYV, ASHN, and SKR populations appear within all phenotypic experiments.

- Supplementary material: In the version I received, the legends are missing, and the table and figures are not numbered. Only two of the figures are referred to in the main text.

Response: We have added figure and table legends in the supplementary material.

Reviewer #2 (Remarks on code availability):

The code is well-documented. It would be helpful to have a README file describing the function of each script. It seems that the script "Demographic_inference.R" is not relevant for this version of the manuscript.

Response: We have removed the script from the GitHub repository and included a README file detailing the function of each of the scripts in the repository. Please see https://github.com/PhDMattyB/Nature_comms

RESPONSE TO REVIEWERS' COMMENTS

Reviewer #1 (Remarks to the Author):

General Comments
Despite the authors' efforts to address previous reviewer comments, specifically in the following five areas:
(1) Addition of common garden experiments and RNA-seq data
(2) PCA analysis of chromosome XXI inversion region
(3) Removal of speculative GO term analysis
(4) Removal of problematic TreeMix analysis
(5) Identification of MAPK pathway in all population pairs
The revisions do not sufficiently resolve the fundamental issues identified in the initial review. Furthermore, several important questions remain inadequately addressed.

1. Limited sample size
A sample size of just four population pairs may not adequately capture the breadth of genetic and phenotypic variation present in both geothermal and surface environments. Different populations can exhibit unique adaptations and evolutionary responses based on their specific ecological contexts, and the restriction to only these pairs limits the generalizability of the results. Each population's ecological history and genetic background can significantly influence adaptive traits. A broader sampling strategy that encompasses more diverse populations from both geothermal and surface habitats would provide a more comprehensive understanding of the adaptive mechanisms at play. With such a small sample size, the statistical analyses performed may lack sufficient power to detect meaningful patterns and differences between groups. Smaller sample sizes can lead to increased variability in results, making it difficult to draw robust conclusions regarding adaptation. In summary, while the study provides intriguing insights into the adaptations of sticklebacks in geothermal environments, the limited representativeness of the chosen population pairs notably constrains the applicability of the findings across broader ecological contexts.

Response

We respectfully disagree and maintain that the sample size does not limit our analyses or the strength of our inferences. With our study design we first build an accurate phylogeny for stickleback diverging along a geothermal-ambient habitat axis that relied on sampling 23 freshwater populations as well as 2 marine populations. This allowed us to identify 4 focal geothermal-ambient population pairs to account for variation in demographic history, population age, phylogenetic distance, and heterogeneity in genomic variation for further in-depth analyses (lines 1073-1084). These four population pairs represented the best candidates for further analyses because they provided some of the most extreme phylogenetic and demographic differences among our larger sampling strategy. In other words, they had the best chance to **show inconsistent results** and provide the greatest opportunity to generalize our results. Our balanced sampling between geothermal and ambient ecotypes allowed for the use of the method to detect parallel allelic divergence (AF-vapeR: https://doi.org/10.1111/2041-210X.13952). As that test itself was recently developed and is designed to work with only 3 replicates, this indicates that novel and core

methods are being developed to handle similar samples sizes that are reflective of the field. In addition, the very nature of testing for parallelism provided by this method is **directly motivated by the ability to generalize findings across populations**. Thus, we believe our results are of high quality with every decision taken to provide biologically real effects.

In terms of the population genomic analyses, we believe we have sufficient statistical power to detect regions of the genome under selection as well as large inversions. Our data encompasses 109 individuals, 173485 biallelic SNPs (after quality filtering), 15-20x coverage of the genome, and a balanced sampling design. Our sample sizes are of similar size and quality to recently published papers in *Nature Communications* (https://doi.org/10.1038/s41467-020-17195-4) and *Nature Ecology and Evolution* (https://doi.org/10.1038/s41559-024-02593-4; https://doi.org/10.1038/s41559-022-01890-0). To further demonstrate where our work fits in, a recently published study on adaptive divergence in *Science* (DOI: 10.1126/science.adp3745) focused on just two divergent mountain refugia.

2. Insufficient functional evidence
Although authors have included common garden experiments and RNA-seq data to support the role of the MAPK signaling pathway, the functional evidence remains weak. Authors have improved the logical connection between genotype and phenotype, but this remains an inferential link rather than a direct demonstration. The connection between the MAPK pathway and the observed phenotypic adaptations is not adequately demonstrated. While the presence of MAPK genes was established, the direct functional implications of these genes in the context of thermal adaptation are still unclear. The lack of direct experimental validation of the proposed mechanisms undermines the strength of conclusions. The authors should acknowledge this limitation explicitly in the Discussion section, framing the mechanistic pathway as a well-supported hypothesis that warrants future experimental validation.

Response

We have now acknowledged this limitation in the discussion of the revised manuscript (line 283-285). In the revised manuscript, we have addressed this point to the satisfaction of reviewer #2 and shown that the *mapk* pathway is repeatedly associated with adaptation to geothermal environments (line 228), shows ecotype-specific plastic gene expression (line 243), and that gene expression patterns are affected by hybridization (line 257), all of which are hallmark signs of adaptation. We provide evidence that the *mapk* pathway plays a role in adaptation to geothermal habitats but we do not currently know what phenotypes are affected. This is a part of ongoing work to validate the role of the *mapk* pathway in geothermal adaptation but is outside the scope of this manuscript.

3. Insufficient evidence for chromosome XXI inversion driving ecological differentiation
The manuscript presents a PCA analysis of the chromosome XXI inversion but does not convincingly demonstrate that this structural variation is responsible for thermal adaptation. The authors acknowledge that the full functional role of the inversion is not understood, and the evidence provided does not adequately support the claim that it facilitates adaptation to

geothermal environments. The analyses presented are insufficient to establish a clear mechanism linking the inversion to ecological differentiation.

Response

As mentioned in the revised manuscript (line 309), this inversion was first identified in Jones et al. (2012) and was suspected to facilitate adaptation to freshwater habitats. This long-standing hypothesis was only recently tested and shown to not be supported (https://doi.org/10.1038/s41437-025-00784-8). In our revised manuscript we included a PCA of the inversion site and shown that the karyotypes differentiate geothermal and ambient ecotypes. We have also shown that the inversion site is a population genomic outlier, has high Fst differentiating geothermal and ambient ecotypes, is under positive recent selection in the geothermal ecotype, and is diverging in parallel across all geothermal-ambient ecotype pairs (Figure 4A-E). The suite of evidence highlights that the functional significance of this inversion may facilitate adaptation to warming environments. The data and evidence that we have provided thus far is in line with other high quality papers in the field such as in *Nature Communications* (https://doi.org/10.1038/s41467-023-36167-y; https://doi.org/10.1038/s41467-021-27173-z; https://doi.org/10.1038/s41467-022-28806-7), *Nature Ecology and Evolution* (https://doi.org/10.1038/s41559-022-01890-0; https://doi.org/10.1038/s41559-024-02593-4), and *Science Advances* (DOI: 10.1126/sciadv.adk7906; DOI: 10.1126/sciadv.aav9963).

4. Limited representation and contribution to the field
While this study presents interesting findings regarding the adaptation of threespine sticklebacks to geothermal habitats, the novelty of the concepts and findings is limited. The authors make a valid point that their system provides insight into populations that have successfully adapted to warming, which is valuable for understanding adaptive potential. However, the magnitude difference between geothermal warming and predicted climate change (order of magnitude difference) remains a legitimate caveat that deserves more explicit acknowledgment. The authors should add a clear statement in the Discussion acknowledging this limitation. For example: "While the temperature differential in our system (~15-20°C) exceeds near-term climate change projections, our findings identify developmental pathways that may represent conserved targets of thermal adaptation. Future studies in systems with more moderate temperature differentials will be important for assessing the generality of these findings."

Response

We have now added in a statement in the discussion acknowledging this limitation (line 428-430). We have also included a section in the discussion entitled ‘A way forward for evolution: the interaction of polygenic adaptation and structural variation’ which specifically highlights the novelty of our manuscript.

5. Speculative interpretation of metabolism and neddylation
The parallel between Icelandic geothermal stickleback and Mexican cavefish is conceptually interesting but the systems differ in important ways. The cavefish system involves extreme, constant resource limitation, whereas the geothermal stickleback system involves seasonal

resource fluctuation with elevated metabolic demands. Clearly label speculative interpretations as hypotheses. Consider adding language such as: "While speculative, the convergent metabolic signatures suggest that thermal adaptation may involve similar physiological solutions across diverse systems, though the specific ecological pressures differ."

Response

We have now added in a statement in the revised discussion (line 343).

The review has titled point 5 as ‘Speculative interpretation of metabolism and neddylation’. However, all references to neddylation were removed in the previous revision. In that revision, we updated the gene ontology analysis and the discussion to ensure that these changes were fully reflected.

In the last round of review we clarified that the only real axis of differentiation between geothermal and ambient habitats is temperature and provided a reference to published environmental data illustrating this point (please see supplementary table 1: https://doi.org/10.1093/evolut/qpac018).

6. Different population combinations across experiments
While logistical constraints are understandable and the statistical consistency across pairs is reassuring, this experimental heterogeneity should be acknowledged as a limitation. The authors have attempted to address the reviewers' concerns regarding statistical analyses, including the use of Fisher’s exact tests and Sorensen-Dice similarity indices. However, the results remain poorly defined, and the statistical methods employed do not adequately support the claims made regarding the significance of outlier loci and their relationship to population age and adaptation. The lack of clear and rigorous statistical testing undermines the reliability of the findings. Add a brief statement in the Methods or Results section acknowledging this limitation. For example: "Due to logistical constraints, different population combinations were used across phenotypic experiments. However, results were statistically consistent across all tested ecotype pairs, and three populations (MYV, ASHN, SKR) appeared in all experiments, providing internal replication."

Response

We have now added in a statement of limitation in the discussion (line 359-362) in line with these concerns.

We have now added another statistical test to evaluate the relationship between outlier loci and genes with a proxy for population age (lines 180-182, 187-189, and 1131-1134). The results show that there is no relationship with the amount of genetic differentiation and the similarity of shared outlier loci or genes. All of the statistics in this manuscript are performed to the highest standards and in line with what has been done in the field (*Nature Communications:* https://doi.org/10.1038/s41467-020-20263-4 and https://doi.org/10.1038/s41467-021-23027-w; *PNAS*: https://doi.org/10.1073/pnas.202271311).

Conclusion
In summary, while the authors have made commendable efforts to address previous reviewer comments, the manuscript ultimately fails to provide sufficient evidence to support its central

claims. The lack of functional evidence, insufficient statistical rigor, and speculative interpretations significantly weaken the study's contributions to the field of evolutionary biology and climate adaptation. Therefore, I recommend rejecting the manuscript in its current form.

Reviewer #2 (Remarks to the Author):

Review for "Parallel adaptation to geothermally-warmed habitats due to common structural variation and functional developmental pathways" by Brachmann et al.

I feel that the authors have addressed the concerns of both the other reviewer and myself and have improved the manuscript. They have clarified important points and strengthened their interpretation of the GO enrichment analysis. In addition, they present further evidence of the role of the inversion on chromosome XXI and common garden experiments that support the role of the mapk signaling pathway in geothermal adaptation. I have a few minor comments regarding the new sections.

Response

We would like the thank the reviewer for acknowledging that the revised manuscript addressed the concerns of both reviewers.

Comments:

- L178-179, Figure 2E: I think the figures work well to compare the levels of parallelism, but I'm unsure how you calculated the predicted degree of genomic parallelism. Please elaborate on this in the methods.

Response

The predicted degree of parallelism figure 2E is a conceptual prediction of the distribution of outlier loci across populations would look like if population age had an effect (in response to reviewer #1). The figure legend of figure 2 has been adjusted to reflect this. The idea is that if divergence time played a role in structuring the number of outlier loci across populations, the younger populations would share more outlier loci with each other than with the older populations. The older populations would share some outliers with each other but have less overlap in general due to adaptation, drift, and erosion of variation due to a longer time since divergence. This figure was based off of results in: https://doi.org/10.1073/pnas.202271311.

- L264-267: I'm having trouble understanding how these Fst values are elevated and point to the braf gene being under selection. These values seem to be close to the average Fst (L164) and don't seem to be classified as outliers in 2A-D.

Response

The Fst values of the *braf* gene are greater than the population average of differentiation in MYV and ASHN but not in SKR and GTS-GAR. We have clarified this in line 270.

- L1169-1174: Why did you decide to use enrichR in addition to Metascape for the gene ontology analysis? I assume that the new results in figure 3B and figure 4F use this method, but this is not clear to me from the manuscript. Please clarify which method you used in each analysis. In addition, what is the meaning of the colors in these figures? In Figure 4F, I find the GO terms harder to understand than before, and I can't quite follow your interpretation that the genes in the inversion are related to metabolism, hypoxia, and cellular repair (L315).

Response

We decided to use enrichR, instead of metascape, for the gene ontology analyses of the genes inside the inversion as enrichR performs better with small candidate gene sets and querying across a broad range of databases (https://doi.org/10.3389/fbinf.2026.1755664). We utilized metascape for the gene ontology analysis for the outlier genes and transcriptomic data as it is designed for large lists of genes, network analysis, and functional theme summarization. The two methods were used as the gene sets differed in size and the goal of the biological interpretation. We have clarified this distinction and justification for these approaches in the methods section (line 1189).

While we agree that the GO terms could be challenging to interpret, we have attempted to alleviate this by distilling them down to the three major biological functions: hypoxia, metabolism, and cellular repair. Hypoxia is related to the hypoxia inducible factor (HIF) pathway. Metabolism is related to the ppary, Beta tako islet cells, SOHLH2 pathway, RARRXR pathway and dichlorobenzene degradation (HSA00627). Lastly, cellular repair is related to the two DNA replication terms (GO1902975 and GO1902315), TERF1 pathway, and ST18 pathway. We have re-coloured Figure 4F and adjusted the figure legend to show the groupings of the enrichR analysis for easier biological interpretation.

- Figure 4B: I think it's helpful to have this figure differentiating the three potential states of the inversion. Please explain what the different shapes mean and the ellipses seem to exclude some samples. However, since the coordinates of the geothermal-ambient inversion overlap with the inversion found in all freshwater populations, I was wondering how you can be sure that the homozygous cluster most common for the geothermal populations is the inverted karyotype and not the ancestral karyotype, while the ambient homozygous cluster is the inversion (L297-304)?

Response

As this figure caused some confusion, we have now added in a statement about what the shapes actually mean in the figure legend for figure 4B. The shapes represent the four geothermal-ambient ecotype pairs and the ellipses are 95% confidence intervals around the k-means clusters. The geothermal cluster is more than likely the inverted karyotype due to a culmination of evidence. This inversion was originally identified in Jones et al (2012) and was suggested to be adaptive in freshwater environments from standing genomic variation in the marine (ancestral form). Most freshwater populations of stickleback are cold/ambient populations at the latitudes encompassing Iceland with only a very few being geothermal. The XP-EHH statistic in Figure 4E shows that the inverted region is under recent selection in the geothermal populations and not the ambient, indicating that the inversion karyotype that the geothermal fish have is the derived and inverted

form of the arrangement. If we only had the PCA results, we could not say which cluster is likely the inverted karyotype. However, the PCA along with all of the other population genomic methods indicates that the geothermal karyotype is more than likely the derived and inverted form as it's under recent selection. We have made this clear in the text at line 297.

- L1098-1011: After removing the Treemix figure, you should also remove the corresponding methods here.

Response

This has been removed from the text.

- Many of the supplementary figures are not mentioned in the main text and I would carefully review them to ensure they are all still relevant for the revised manuscript.

Response

We would like to thank the reviewer for the suggestion. We have now referred to the relevant supplementary files in the main body of the text.

- L1239-1251: The methods seem to be very similar for both common garden experiments, so I would remove this and mention that it was performed as described above.

Response

We have opted to keep all of the methods for the common garden experiments separated to improve clarity. While there are a lot of similarities in the methods, there are a couple of key differences. The first common garden experiment was performed on geothermal and ambient ecotypes from three independent population pairs and the liver was sequenced. For the second common garden experiment, hybrids were made from one specific population pair (SKR) and the brain was sequenced. As the two methods had a different number of populations, the analytical approaches were completely different. In the first experiment, we used a GLM to model gene expression and account for family effects. Whiles in the second, we used LIMMA to understand expression across specific biological contrasts. We wanted to make sure that the differences between the experiments were very clear, repeatable, and not mislead the results. However, if the reviewer feels strongly about this, we can amend if necessary.

- Figure 6 should be labelled as figure 5.

Response

This has now been corrected.
